# Supplementary figures and images for: Identification of evolutionary relationships and DNA markers in the medicinally important genus Fritillaria based on chloroplast genomics
Source: PeerJ. 2021 Dec 16;9:e12612. doi: 10.7717/peerj.12612 (PMC8684722; doi:10.7717/peerj.12612)

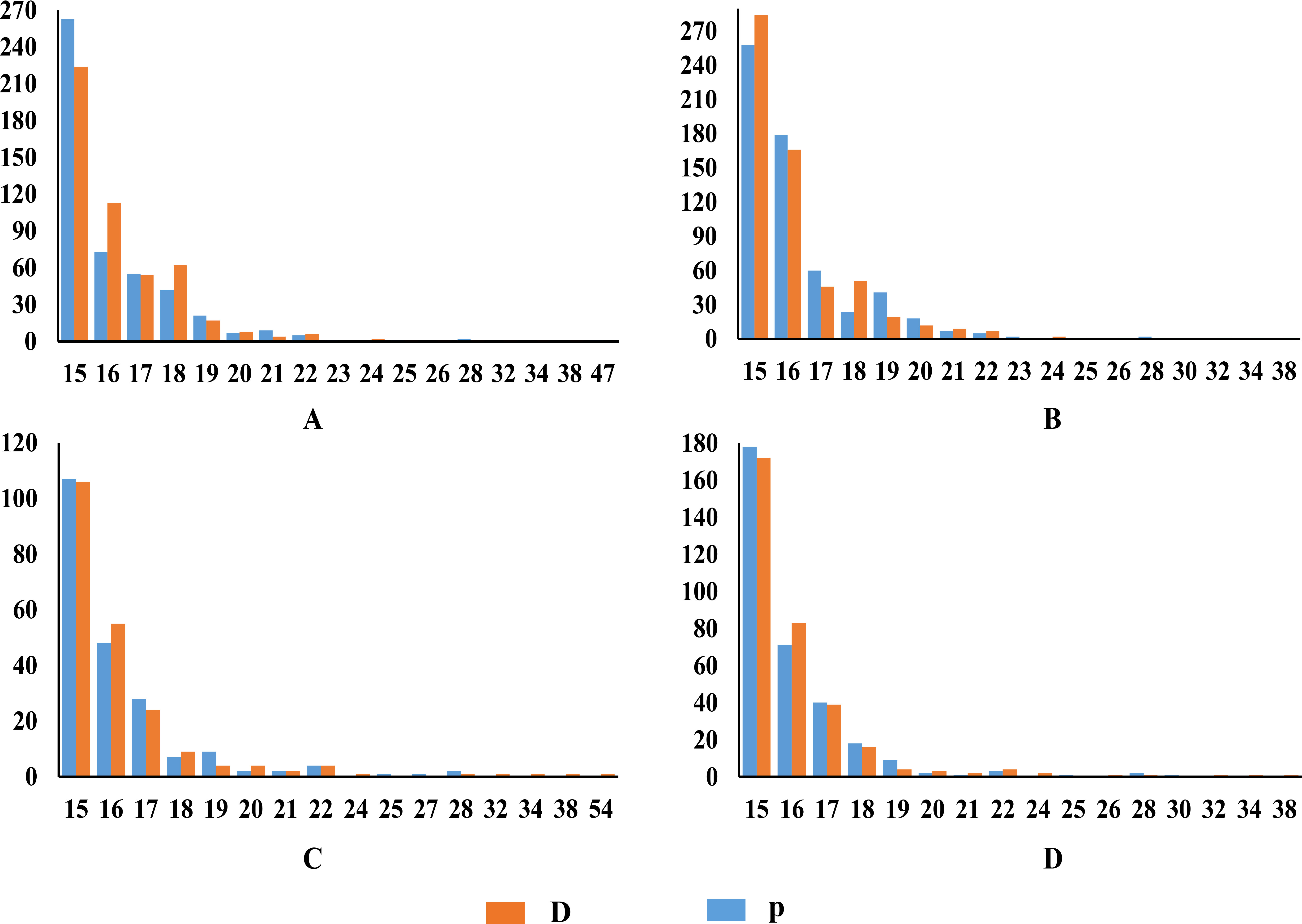

Supplement: Supplemental Information 1 — A Fritillaria unibracteata repeat sequence. B Fritillaria przewalskii repeat sequence. C Fritillaria delavayi repeat sequence. D Fritillaria sinica repeat sequence. Abscissa is the type of scattered repetition sequence, and ordinate is the number of scattered repetition sequence. D represents positive repetition, P represents palindrome repetition (including reverse and complementary). [file peerj-09-12612-s001.png]

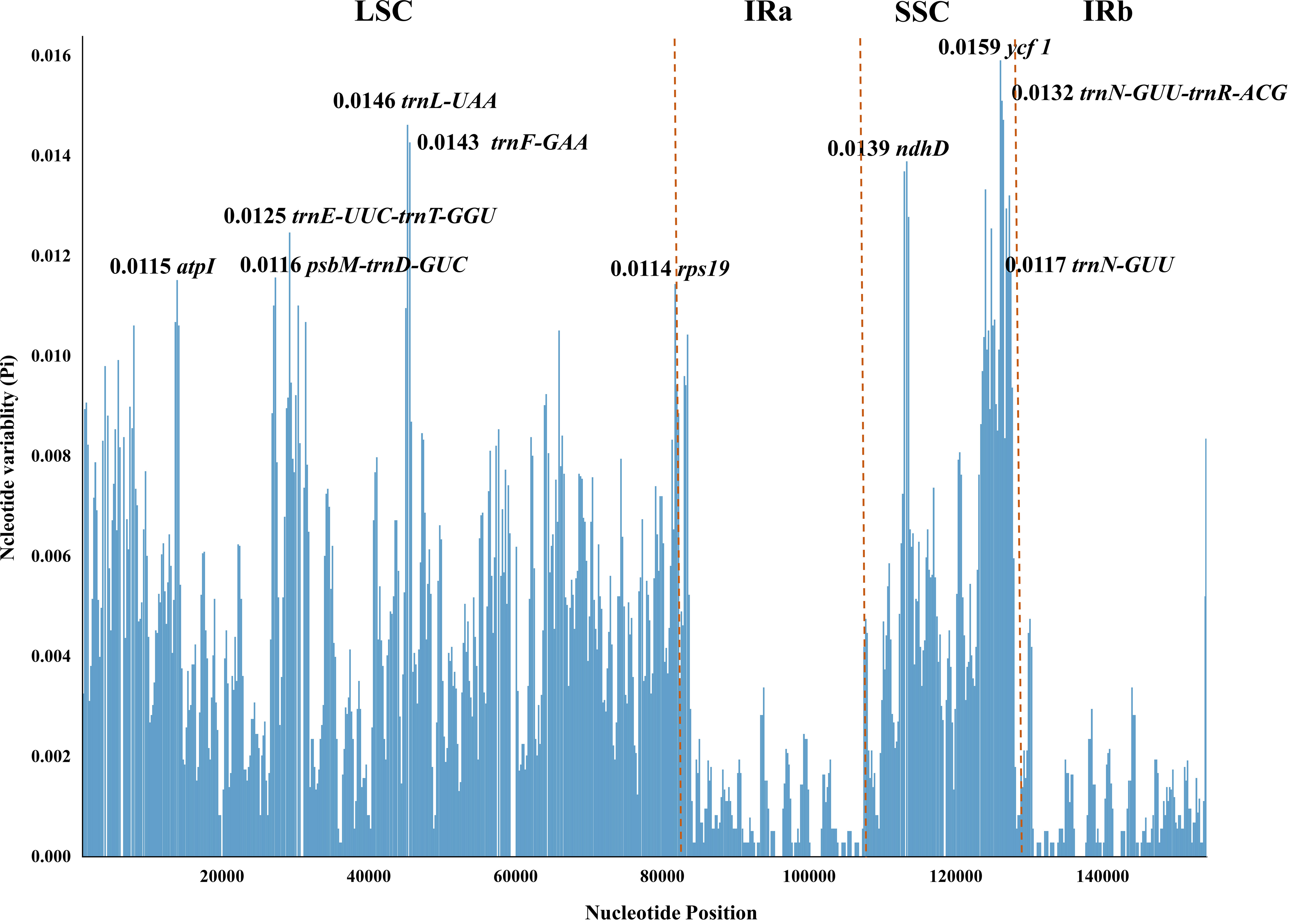

Supplement: Supplemental Information 2 — (Window length: 600 bp, step size: 200 bp). X-axis indicated the position of the midpoint of a window. Y-axis indicated the nucleotide diversity of each window. [file peerj-09-12612-s002.png]

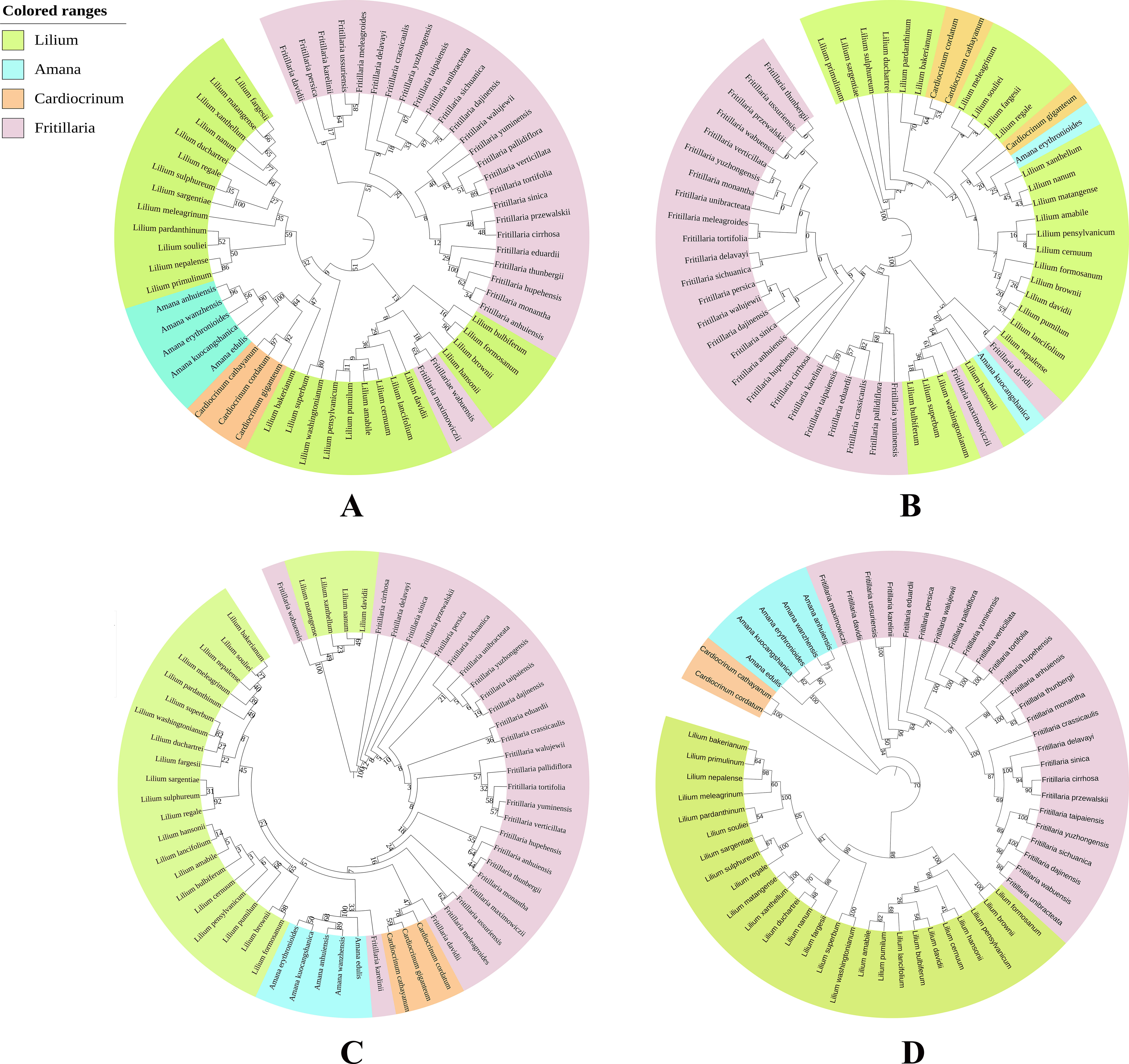

Supplement: Supplemental Information 4 — Numbers above nodes are supporting values with NJ bootstrap values. A: phylogenetic analysis of matK sequence; B: phylogenetic analysis of psbA-trnH sequence; C: phylogenetic analysis of rpl16 sequence; D: phylogenetic analysis of ycf1 sequence. [file peerj-09-12612-s004.png]

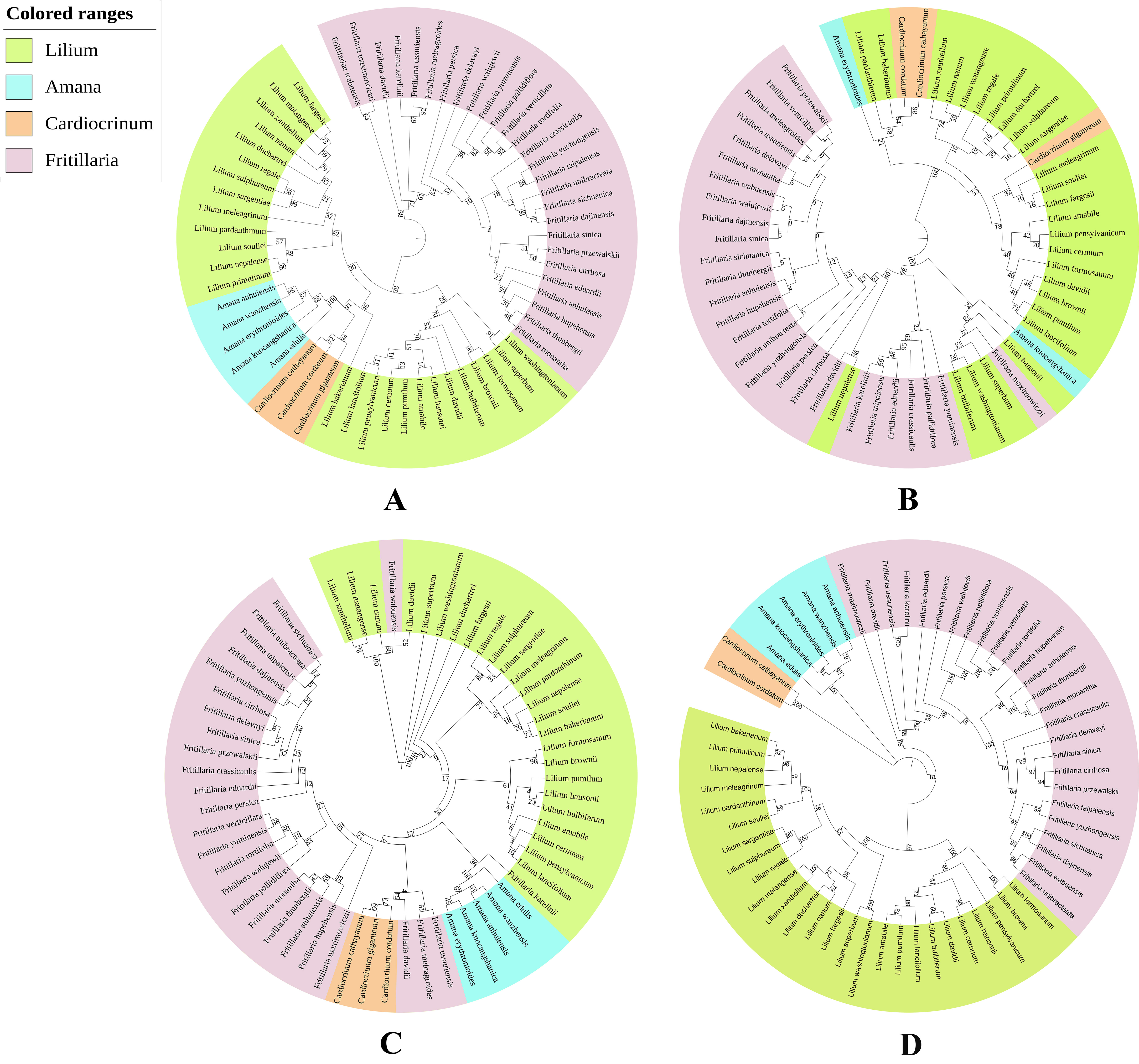

Supplement: Supplemental Information 5 — Numbers above nodes are supporting values with NJ bootstrap values. A: phylogenetic analysis of matK sequence; B: phylogenetic analysis of psbA-trnH sequence; C: phylogenetic analysis of rpl16 sequence; D: phylogenetic analysis of ycf1 sequence. [file peerj-09-12612-s005.png]

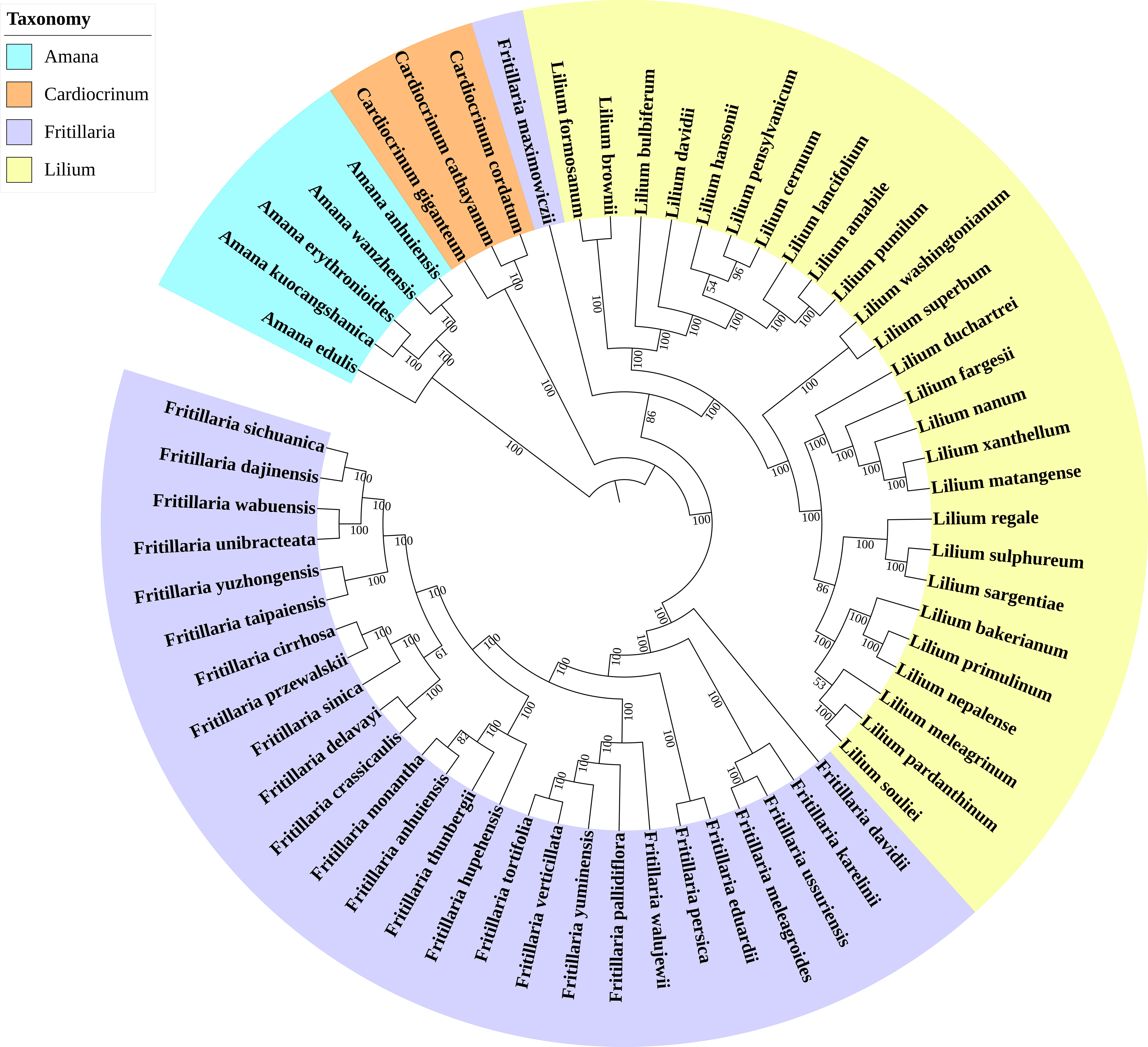

Supplement: Supplemental Information 6 — Numbers above nodes are supporting values with NJ bootstrap values. [file peerj-09-12612-s006.png]
