# Supplementary material for: Identification of evolutionary relationships and DNA markers in the medicinally important genus Fritillaria based on chloroplast genomics
Source: PeerJ. 2021 Dec 16;9:e12612. doi: 10.7717/peerj.12612 (PMC8684722; doi:10.7717/peerj.12612)
Supplement: Supplemental Information 3 — The potential species-specific molecular markers were represented by squares and rectangles for F. ussuriensis (black), F.pallidiflora (blue), F. thunbergii (green), F. taipaiensis (purple), F. walujewii (light green), F.delavayi (dark blue), F. hupehensis (orange), F. unibracteata (pink) and F. wabuensis (dark red), respectively. [file peerj-09-12612-s003.pdf]

```
F.unibracteata   ATGATTTTGAAATCTTTTCTACTAGGTAATCTATTATCCTTATGCATGAAGATAATAAATTCGGTCGTTGTGGTC   75
F.przewalskii    ATGATTTTGAAATCTTTTCTACTAGGTAATCTATTATCCTTATGCATGAAGATAATAAATTCGGTCGTTGTGGTC   75
F.hupehensis     ATGATTTTGAAATCTTTTCTACTAGGTAATCTATTATCCTTATGCATGAAGATAATAAATTCGGTCGTTGTGGTC   75
F.cirrhosa       ATGATTTTGAAATCTTTTCTACTAGGTAATCTATTATCCTTATGCATGAAGATAATAAATTCGGTCGTTGTGGTC   75
F.ussuriensis    ATGATTTTGAAATCTTTTCTACTAGGTAATCTATTATCCTTATGCATGAAGATAATAAATTCGGTCGTTGTGGTC   75
F.delavayi       ATGATTTTGAAATCTTTTCTACTAGGTAATCTATTATCCTTATGCATGAAGATAATAAATTCGGTCGTTGTGGTC   75
F.taipaiensis    ATGATTTTGAAATCTTTTCTACTAGGTAATCTATTATCCTTATGCATGAAGATAATAAATTCGGTCGTTGTGGTC   75
F.wabuensis      ATGATTTTGAAATCTTTTCTACTAGGTAATCTATTATCCTTATGCATGAAGATAATAAATTCGGTCGTTGTGGTC   75
F.walujewii      ATGATTTTGAAATCTTTTCTACTAGGTAATCTATTATCCTTATGCATGAAGATAATAAATTCGGTCGTTGTGGTC   75
F.pallidiflora   ATGATTTTGAAATCTTTTCTACTAGGTAATCTATTATCCTTATGCATGAAGGTAATAAATTCGGTCGTTGTGGTC   75
F.thunbergii     ATGATTTTGAAATCTTTTCTACTAGGTAATCTATTATCCTTATGCATGAAGATAATAAATTCGGTCGTTGTGGTC   75

F.unibracteata   GGACTCTATTATGGATTTCTGACCACATTCTCCATAGGGCCCTCTTATCTCTTCCTTCTCCGAGCTCGGGTTATG   150
F.przewalskii    GGACTCTATTATGGATTTCTGACCACATTCTCCATAGGGCCCTCTTATCTCTTCCTTCTCCGAGCTCGGGTTATG   150
F.hupehensis     GGACTCTATTATGGATTTCTGACCACATTCTCCATAGGGCCCTCTTATCTCTTCCTTCTCCGAGCTCGGGTTATG   150
F.cirrhosa       GGACTCTATTATGGATTTCTGACCACATTCTCCATAGGGCCCTCTTATCTCTTCCTTCTCCGAGCTCGGGTTATG   150
F.ussuriensis    GGACTCTATTATGGATTTCTGACCACATTCTCCATAGGGCCCTCTTATCTCTTCCTTCTCCGAGCTCGGGTTATG   150
F.delavayi       GGACTCTATTATGGATTTCTGACCACATTCTCCATAGGGCCCTCTTATCTCTTCCTTCTCCGAGCTCGGGTTATG   150
F.taipaiensis    GGACTCTATTATGGATTTCTGACCACATTCTCCATAGGGCCCTCTTATCTCTTCCTTCTCCGAGCTCGGGTTATG   150
F.wabuensis      GGACTCTATTATGGATTTCTGACCACATTCTCCATAGGGCCCTCTTATCTCTTCCTTCTCCGAGCTCGGGTTATG   150
F.walujewii      GGACTCTATTATGGATTTCTGACCACATTCTCCATAGGGCCCTCTTATCTCTTCCTTCTCCGAGCTCGGGTTATG   150
F.pallidiflora   GGACTCTATTATGGATTTCTGACCACATTCTCCATAGGGCCCTCTTATCTCTTCCTTCTCCGAGCTCGGGTTATG   150
F.thunbergii     GGACTCTATTATGGATTTCTGACCACATTCTCCATAGGGCCCTCTTATCTCTTCCTTCTCCGAGCTCGGGTTATG   150

F.unibracteata   GAAGAAGGAACCGAGAAGGGGGTATCAGCAACAACTGGTTTTATTACGGGACAGCTCATGATGTTCATATCGATC   225
F.przewalskii    GAAGAAGGAACCGAGAAGGGGGTATCAGCAACAACTGGTTTTATTACGGGACAGCTCATGATGTTCATATCGATC   225
F.hupehensis     GAAGAAGGAACCGAGAAGGGGGTATCAGCAACAACTGGTTTTATTACGGGACAGCTCATGATGTTCATATCGATC   225
F.cirrhosa       GAAGAAGGAACCGAGAAGGGGGTATCAGCAACAACTGGTTTTATTACGGGACAGCTCATGATGTTCATATCGATC   225
F.ussuriensis    GAAGAAGGAACCGAGAAGGGGGTATCAGCAACAACGGGTTTTATTACGGGACAGCTCATGATGTTCATATCGATC   225
F.delavayi       GAAGAAGGAACCGAGAAGGGGGTATCAGCAACAACTGGTTTTATTACGGGACAGCTCATGATGTTCATATCGATC   225
F.taipaiensis    GAAGAAGGAACCGAGAAGGGGGTATCAGCAACAACTGGTTTTATTACGGGACAGCTCATGATGTTCATATCGATC   225
F.wabuensis      GAAGAAGGAACCGAGAAGGGGGTATCAGCAACAACTGGTTTTATTACGGGACAGCTCATGATGTTCATATCGATC   225
F.walujewii      GAAGAAGGAACCGAGAAGGGGGTATCAGCAACAACTGGTTTTATTACGGGACAGCTCATGATGTTCATATCGATC   225
F.pallidiflora   GAAGAAGGAACCGAGAAGGGGGTATCAGCAACAACTGGTTTTATTACGGGACAGCTCATGATGTTCATATCGATC   225
F.thunbergii     GAAGAAGGAACCGAGAAGGGGGTATCAGCAACAACTGGTTTTATTACGGGACAGCTCATGATGTTCATATCGATC   225

F.unibracteata   TATTATGCGCCTCTGCATCTAGCATTGGGTAGACCTCATACAATAACTGTCCTAGTTCTACCGTATCTTTTGTTT   300
F.przewalskii    TATTATGCGCCTCTGCATCTAGCATTGGGTAGACCTCATACAATAACTGTCCTAGTTCTACCGTATCTTTTGTTT   300
F.hupehensis     TATTATGCGCCTCTGCATCTAGCATTGGGTAGACCTCATACAATAACTGTCCTAGTTCTACCGTATCTTTTGTTT   300
F.cirrhosa       TATTATGCGCCTCTGCATCTAGCATTGGGTAGACCTCATACAATAACTGTCCTAGTTCTACCGTATCTTTTGTTT   300
F.ussuriensis    TATTATGCGCCTCTGCATCTAGCATTGGGTAGACCTCATACAATAACTGTCCTAGTTCTACCGTATCTTTTGTTT   300
F.delavayi       TATTATGCGCCTCTGCATCTAGCATTGGGTAGACCTCATACAATAACTGTCCTAGTTCTACCGTATCTTTTGTTT   300
F.taipaiensis    TATTATGCGCCTCTGCATCTAGCATTGGGTAGACCTCATACAATAACTGTCCTAGTTCTACCGTATCTTTTGTTT   300
F.wabuensis      TATTATGCGCCTCTGCATCTAGCATTGGGTAGACCTCATACAATAACTGTCCTAGTTCTACCGTATCTTTTGTTT   300
F.walujewii      TATTATGCGCCTCTGCATCTAGCATTGGGTAGACCTCATACAATAACTGTCCTAGTTCTACCGTATCTTTTGTTT   300
F.pallidiflora   TATTATGCGCCTCTGCATCTAGCATTGGGTAGACCTCATACAATAACTGTCCTAGTTCTACCGTATCTTTTGTTT   300
F.thunbergii     TATTATGCGCCTCTGCATCTAGCATTGGGTAGACCTCATACAATAACTGTCCTAGTTCTACCGTATCTTTTGTTT   300

F.unibracteata   CATTTCTTCTGGAACAATCACAAAAACCTTTTTGATTATGGATCTACTACCAGAAATTCAATGCGTAATCTCAGC   375
F.przewalskii    CATTTCTTCTGGAACAATCACAAAAACCTTTTTGATTATGGATCTACTACCAGAAATTCAATGCGTAATCTCAGC   375
F.hupehensis     CATTTCTTCTGGAACAATCACAAAAACCTTTTTGATTATGGATCTACTACCAGAAATTCAATGCGTAATCTCAGC   375
F.cirrhosa       CATTTCTTCTGGAACAATCACAAAAACCTTTTTGATTATGGATCTACTACCAGAAATTCAATGCGTAATCTCAGC   375
F.ussuriensis    CATTTCTTCTGGAACAATCACAAAAACCTTTTTGATTATGGATCTACTACCAGAAATTCAATGCGTAATCTCAGC   375
F.delavayi       CATTTCTTCTGGAACAATCACAAAAACCTTTTTGATTATGGATCTACTACCAGAAATTCAATGCGTAATCTCAGC   375
F.taipaiensis    CATTTCTTCTGGAACAATCACAAAAACCTTTTTGATTATGGATCTACTACCAGAAATTCAATGCGTAATCTCAGC   375
F.wabuensis      CATTTCTTCTGGAACAATCACAAAAACCTTTTTGATTATGGATCTACTACCAGAAATTCAATGCGTAATCTCAGC   375
F.walujewii      CATTTCTTCTGGAACAATCACAAAAACCTTTTTGATTATGGATCTACTACCAGAAATTCAATGCGTAATCTCAGC   375
F.pallidiflora   CATTTCTTCTGGAACAATCACAAAAACCTTTTTGATTATGGATCTACTACCAGAAATTCAATGCGTAATTTCAGC   375
F.thunbergii     CATTTCTTCTGGAACAATCACAAAAACCTTTTTGATTATGGATCTACTACCAGAAATTCAATGCGTAATCTCAGC   375

F.unibracteata   ATTCAATGTGTATTCCTGAATAATCTAATTTTTCAATTATTCAACCATTTCATTTTACCAAGCTCAACGTTAACC   450
F.przewalskii    ATTCAATGTGTATTCCTGAATAATCTAATTTTTCAATTATTCAACCATTTCATTTTACCAAGCTCAACGTTAACC   450
F.hupehensis     ATTCAATGTGTATTCCTGAATAATCTAATTTTTCAATTATTCAACCATTTCATTTTACCAAGCTCAACGTTAACC   450
F.cirrhosa       ATTCAATGTGTATTCCTGAATAATCTAATTTTTCAATTATTCAACCATTTCATTTTACCAAGCTCAACGTTAACC   450
F.ussuriensis    ATTCAATGTGTATTCCTGAATAATCTAATTTTTCAATTATTCAACCATTTCATTTTACCAAGCTCAACGTTAACC   450
F.delavayi       ATTCAATGTGTATTCCTGAATAATCTAATTTTTCAATTATTCAACCATTTCATTTTACCAAGCTCAACGTTAACC   450
F.taipaiensis    ATTCAATGTGTATTCCTGAATAATCTAATTTTTCAATTATTCAACCATTTCATTTTACCAAGCTCAACGTTAACC   450
F.wabuensis      ATTCAATGTGTATTCCTGAATAATCTAATTTTTCAATTATTCAACCATTTCATTTTACCAAGCTCAACGTTAACC   450
F.walujewii      ATTCAATGTGTATTCCTGAATAATCTAATTTTTCAATTATTCAACCATTTCATTTTACCAAGCTCAACGTTAACC   450
F.pallidiflora   ATTCAATGTGTATTCCTGAATAATCTAATTTTTCAATTATTCAACCATTTCATTTTACCAAGCTCAACGTTAACC   450
F.thunbergii     ATTCAATGTGTATTCCTGAATAATCTAATTTTTCAATTATTCAACCATTTCATTTTACCAAGCTCAACGTTAACC   450

F.unibracteata   AGATTAGTCAACATTTATACGTTTCGATGCAACAATAAGATGTTATTTGTAACAAGTAGTTTTGTTGGTTGGTTA   525
F.przewalskii    AGATTAGTCAACATTTATACGTTTCGATGCAACAATAAGATGTTATTTGTAACAAGTAGTTTTGTTGGTTGGTTA   525
F.hupehensis     AGATTAGTCAACATTTATACGTTTCGATGCAACAATAAGATGTTATTTGTAACAAGTAGTTTTGTTGGTTGGTTA   525
F.cirrhosa       AGATTAGTCAACATTTATACGTTTCGATGCAACAATAAGATGTTATTTGTAACAAGTAGTTTTGTTGGTTGGTTA   525
F.ussuriensis    AGATTAGTCAACATTTATACGTTTCGATGCAACAATAAGATGTTATTTGTAACAAGTAGTTTTGTTGGTTGGTTA   525
F.delavayi       AGATTAGTCAACATTTATACGTTTCGATGCAACAATAAGATGTTATTTGTAACAAGTAGTTTTGTTGGTTGGTTA   525
F.taipaiensis    AGATTAGTCAACATTTATACGTTTCGATGCAACAATAAGATGTTATTTGTAACAAGTAGTTTTGTTGGTTGGTTA   525
F.wabuensis      AGATTAGTCAACATTTATACGTTTCGATGCAACAATAAGATGTTATTTGTAACAAGTAGTTTTGTTGGTTGGTTA   525
F.walujewii      AGATTAGTCAACATTTATACGTTTCGATGCAACAATAAGATGTTATTTGTAACAAGTAGTTTTGTTGGTTGGTTA   525
F.pallidiflora   AGATTAGTCAACATTTATACGTTTCGATGCAACAATAAGATGTTATTTGTAACAAGTAGTTTTGTTGGTTGGTTA   525
F.thunbergii     AGATTAGTCAACATTTATACGTTTCGATGCAACAATAAGATGTTATTTGTAACAAGTAGTTTTGTTGGTTGGTTA   525
```

```
F.unibracteata   ATTGGTCACATTTTATTCATGAAATGGGTTGGATTGGTCTTAGTCTGGATACGGCAAAATCATTCTATTCGATCT   600
F.przewalskii    ATTGGTCACATTTTATTCATGAAATGGGTTGGATTGGTCTTAGTCTGGATACGGCAAAATCATTCTATTCGATCT   600
F.hupehensis     ATTGGTCACATTTTATTCATGAAATGGGTTGGATTGGTCTTAGTCTGGATACGGCAAAATCATTCTATTCGATCT   600
F.cirrhosa       ATTGGTCACATTTTATTCATGAAATGGGTTGGATTGGTCTTAGTCTGGATACGGCAAAATCATTCTATTCGATCT   600
F.ussuriensis    ATTGGTCACATTTTATTCATGAAATGGGTTGGATTGGTCTTAGTCTGGATACGGCAAAATCATTCTATTCGATCT   600
F.delavayi       ATTGGTCACATTTTATTCATGAAATGGGTTGGATTGGTCTTAGTCTGGATACGGCAAAATCATTCTATTCGATCT   600
F.taipaiensis    ATTGGTCACATTTTATTCATGAAATGGGTTGGATTGGTCTTAGTCTGGATACGGCAAAATCATTCTATTCGATCT   600
F.wabuensis      ATTGGTCACATTTTATTCATGAAATGGGTTGGATTGGTCTTAGTCTGGATACGGCAAAATCATTCTATTCGATCT   600
F.walujewii      ATTGGTCACATTTTATTCATGAAATGGGTTGGATTGGTCTTAGTCTGGATACGGCAAAATCATTCTATTCGATCT   600
F.pallidiflora   ATTGGTCACATTTTATTCATGAAATGGGTTGGATTGGTCTTAGTCTGGATACGGCAAAATCATTCTATTCGATCT   600
F.thunbergii     ATTGGTCACATTTTATTCATGAAATGGGTTGGATTGGTCTTAGTCTGGATACGGCAAAATCATTCTATTCGATCT   600

F.unibracteata   AATAAGTACCTTGTGTCAGAATTGAGAAATTATATGGCTCGAATCTTTAGTATTCTCTTATTTATCACCTGTGTC   675
F.przewalskii    AATAAGTACCTTGTGTCAGAATTGAGAAATTATATGGCTCGAATCTTTAGTATTCTCTTATTTATCACCTGTGTC   675
F.hupehensis     AATAAGTACCTTGTGTCAGAATTGAGAAATTATATGGCTCGAATCTTTAGTATTCTCTTATTTATCACCTGTGTC   675
F.cirrhosa       AATAAGTACCTTGTGTCAGAATTGAGAAATTATATGGCTCGAATCTTTAGTATTCTCTTATTTATCACCTGTGTC   675
F.ussuriensis    AATAAGTACCTTGTGTCAGAATTGAGAAATTATATGGCTCGAATCTTTAGTATTCTCTTATTTATCACCTGTGTC   675
F.delavayi       AATAAGTACCTTGTGTCAGAATTGAGAAATTATATGGCTCGAATCTTTAGTATTCTCTTATTTATCACCTGTGTC   675
F.taipaiensis    AATAAGTACCTTGTGTCAGAATTGAGAAATTATATGGCTCGAATCTTTAGTATTCTCTTATTTATCACCTGTGTC   675
F.wabuensis      AATAAGTACCTTGTGTCAGAATTGAGAAATTATATGGCTCGAATCTTTAGTATTCTCTTATTTATCACCTGTGTC   675
F.walujewii      AATAAGTACCTTGTGTCAGAATTGAGAAATTATATGGCTCGAATCTTTAGTATTCTCTTATTTATCACCTGTGTC   675
F.pallidiflora   AATAAGTACCTTGTGTCAGAATTGAGAAATTATATGGCTCGAATCTTTAGTATTCTCTTATTTATCACCTGTGTC   675
F.thunbergii     AATAAGTACCTTGTGTCAGAATTGAGAAATTATATGGCTCGAATCTTTAGTATTCTCTTATTTATCACCTGTGTC   675

F.unibracteata   TACTATTTAGGCAGAATGCCGTCGCCTATTGTCACTAAGAAACTGAAAGAAACCTCAGAAACAGAAGAAAAGGGA   750
F.przewalskii    TACTATTTAGGCAGAATGCCGTCGCCTATTGTCACTAAGAAACTGAAAGAAACCTCAGAAACAGAAGAAAAGGGA   750
F.hupehensis     TACTATTTAGGCAGAATGCCGTCGCCTATTGTCACTAAGAAACTGAAAGAAACCTCAGAAACAGAAGAAAAGGGA   750
F.cirrhosa       TACTATTTAGGCAGAATGCCGTCGCCTATTGTCACTAAGAAACTGAAAGAAACCTCAGAAACAGAAGAAAAGGGA   750
F.ussuriensis    TACTATTTAGGCAGAATGCCGTCGCCTATTATCACTAAGAAACTGAAAGAAACCTCAGAAACAGAAGAAAAGGGA   750
F.delavayi       TACTATTTAGGCAGAATGCCGTCGCCTATTGTCACTAAGAAACTGAAAGAAACCTCAGAAACAGAAGAAAAGGGA   750
F.taipaiensis    TACTATTTAGGCAGAATGCCGTCGCCTATTGTCACTAAGAAACTGAAAGAAACCTCAGAAACAGAAGAAAAGGGA   750
F.wabuensis      TACTATTTAGGCAGAATGCCGTCGCCTATTGTCACTAAGAAACTGAAAGAAACCTCAGAAACAGAAGAAAAGGGA   750
F.walujewii      TACTATTTAGGCAGAATGCCGTCGCCTATTGTCACTAAGAAACTGAAAGAAACCTCAGAAACAGAAGAAAAGGGA   750
F.pallidiflora   TACTATTTAGGCAGAATGCCGTCGCCTATTGTCACTAAGAAACTGAAAGAAACCTCAGAAACAGAAGAAAAGGGA   750
F.thunbergii     TACTATTTAGGCAGAATGCCGTCGCCTATTGTCACTAAGAAACTGAAAGAAACCTCAGAAACAGAAGAAAAGGGA   750

F.unibracteata   GAAAGTGAGGAAGAAATCGATGTAGAAACAACTTCCGAAACGAAGGCGACTAAACAGGAACAAGGGGGATCCACC   825
F.przewalskii    GAAAGTGAGGAAGAAATCGATGTAGAAACAACTTCCGAAACGAAGGCGACTAAACAGGAACAAGGGGGATCCACC   825
F.hupehensis     GAAAGTGAGGAAGAAATCGATGTAGAAACAACTTCCGAAACGAAGGCGACTAAACAGGAACAAGGGGGATCCACC   825
F.cirrhosa       GAAAGTGAGGAAGAAATCGATGTAGAAACAACTTCCGAAACGAAGGCGACTAAACAGGAACAAGGGGGATCCACC   825
F.ussuriensis    GAAAGTGAGGAAGAAATCGATGTAGAAACAACTTCTGAAACGAAGGCGACTAAACAGGAACAAGGGGGATCCACC   825
F.delavayi       GAAAGTGAGGAAGAAATCGATGTAGAAACAACTTCCGAAACGAAGGCGACTAAACAGGAACAAGGGGGATCCACC   825
F.taipaiensis    GAAAGTGAGGAAGAAATCGATGTAGAAACAACTTCCGAAACGAAGGCGACTAAACAGGAACAAGGGGGATCCACC   825
F.wabuensis      GAAAGTGAGGAAGAAATCGATGTAGAAACAACTTCCGAAACGAAGGCGACTAAACAGGAACAAGGGGGATCCACC   825
F.walujewii      GAAAGTGAGGAAGAAATCGATGTAGAAACAACTTCCGAAACGAAGGCGACTAAACAGGAACAAGGGGGATCCACC   825
F.pallidiflora   GAAAGTGAGGAAGAAATCGATGTAGAAACAACTTCCGAAACGAAGGCGACTAAACAGGAACAAGGGGGATCCACC   825
F.thunbergii     GAAAGTGAGGAAGAAATCGATGTAGAAACAACTTCCGAAACGAAGGCGACTAAACAGGAACAAGGGGGATCCACC   825

F.unibracteata   GAAGAAGACCCTTCCCTTTGTTCGGAAGAAAAAGAGGATCCGGACAAAATAGATGAAACGGAAGAGATCCGGGTG   900
F.przewalskii    GAAGAAGACCCTTCCCTTTGTTCGGAAGAAAAAGAGGATCCGGACAAAATAGATGAAACGGAAGAGATCCGGGTG   900
F.hupehensis     GAAGAAGACCCTTCCCTTTGTTCGGAAGAAAAAGAGGATCCGGACAAAATAGATGAAACGGAAGAGATCCGGGTG   900
F.cirrhosa       GAAGAAGACCCTTCCCTTTGTTCGGAAGAAAAAGAGGATCCGGACAAAATAGATGAAACGGAAGAGATCCGGGTG   900
F.ussuriensis    GAAGAAGACCCTTCCCTTTGTTCGGAAGAAAAAGAGGATCCGGACAAAATAGATGAAACGGAAGAGATCCGGGTG   900
F.delavayi       GAAGAAGACCCTTCCCTTTGTTCGGAAGAAAAAGAGGATCCGGACAAAATAGATGAAACGGAAGAGATCCGGGTG   900
F.taipaiensis    GAAGAAGACCCTTCCCTTTGTTCGGAAGAAAAAGAGGATCCGGACAAAATAGATGAAACGGAAGAGATCCGGGTG   900
F.wabuensis      GAAGAAGACCCTTCCCTTTGTTCGGAAGAAAAAGAGGATCCGGACAAAATAGATGAAACGGAAGAGATCCGGGTG   900
F.walujewii      GAAGAAGACCCTTCCCTTTGTTCGGAAGAAAAAGAGGATCCGGACAAAATAGATGAAACGGAAGAGATCCGGGTG   900
F.pallidiflora   GAAGAAGACCCTTCCCTTTGTTCGGAAGAAAAAGAGGATCCGGACAAAATAGATGAAACGGAAGAGATCCGGGTG   900
F.thunbergii     GAAGAAGACCCTTCCCTTTGTTCGGAAGAAAAAGAGGATCCGGACAAAATAGATGAAACGGAAGAGATCCGGGTG   900

F.unibracteata   AATGGAAAGGAAAAAACAAAAGATGAATTCCACTTTAAAGAGACATCCTATAAGGATAGCCCTGTTGACGAAGAT   975
F.przewalskii    AATGGAAAGGAAAAAACAAAAGATGAATTCCACTTTAAAGAGACATCCTATAAGGATAGCCCTGTTGACGAAGAT   975
F.hupehensis     AATGGAAAGGAAAAAACAAAAGATGAATTCCACTTTAAAGAGACATCCTATAAGGATAGCCCTGTTGACGAAGAT   975
F.cirrhosa       AATGGAAAGGAAAAAACAAAAGATGAATTCCACTTTAAAGAGACATCCTATAAGGATAGCCCTGTTGACGAAGAT   975
F.ussuriensis    AATGGAAAGGAAAAAACAAAAGATGAATTCCACTTTAAAGAGACATCCTATAAGGATAGCCCTGTTGACGAAGAT   975
F.delavayi       AATGGAAAGGAAAAAACAAAAGATGAATTCCACTTTAAAGAGACATCCTATAAGGATAGCCCTGTTGACGAAGAT   975
F.taipaiensis    AATGGAAAGGAAAAAACAAAAGATGAATTCCACTTTAAAGAGACATCCTATAAGGATAGCCCTGTTGACGAAGAT   975
F.wabuensis      AATGGAAAGGAAAAAACAAAAGATGAATTCCACTTTAAAGAGACATCCTATAAGGATAGCCCTGTTGACGAAGAT   975
F.walujewii      AATGGAAAGGAAAAAACAAAAGATGAATTCCACTTTAAAGAGACATCCTATAAGGATAGCCCTGTTGACGAAGAT   975
F.pallidiflora   AATGGAAAGGAAAAAACAAAAGATGAATTCCACTTTAAAGAGACATCCTATAAGGATAGCCCTGTTGACGAAGAT   975
F.thunbergii     AATGGAAAGGAAAAAACAAAAGATGAATTCCACTTTAAAGAGACATCCTATAAGGATAGCCCTGTTGACGAAGAT   975

F.unibracteata   TCTTATCTTGATGGGTATCAAGAGAATTGGGAATTGGGAAGACTTAAAGAAGAAAAAAAAGAAAAGACCCATGCC   1050
F.przewalskii    TCTTATCTTGATGGGTATCAAGAGAATTGGGAATTGGGAAGACTTAAAGAAGAAAAAAAAGAAAAGACCCATGCC   1050
F.hupehensis     TCTTATCTTGATGGGTATCAAGAGAATTGGGAATTGGGAAGACTTAAAGAAGAAAAAAAAGAAAAGACCCATGCC   1050
F.cirrhosa       TCTTATCTTGATGGGTATCAAGAGAATTGGGAATTGGGAAGACTTAAAGAAGAAAAAAAAGAAAAGACCCATGCC   1050
F.ussuriensis    TCTTATCTTGATGGGTATCAAGAGAATTGGGAATTGGGAAGACTTAAAGAAGAAAAAAAAGAAAAGACCCATGCC   1050
F.delavayi       TCTTATCTTGATGGGTATCAAGAGAATTGGGAATTGGGAAGACTTAAAGAAGAAAAAAAAGAAAAGACCCATGCC   1050
F.taipaiensis    TCTTATCTTGATGGGTATCAAGAGAATTGGGAATTGGGAAGACTTAAAGAAGAAAAAAAAGAAAAGACCCATGCC   1050
F.wabuensis      TCTTATCTTGATGGGTATCAAGAGAATTGGGAATTGGGAAGACTTAAAGAAGAAAAAAAAGAAAAGACCCATGCC   1050
F.walujewii      TCTTATCTTGATGGGTATCAAGAGAATTGGGAATTGGGAAGACTTAAAGAAGAAAAAAAAGAAAAGACCCATGCC   1050
F.pallidiflora   TCTTATCTTGATGGGTATCAAGAGAATTGGGAATTGGGAAGACTTAAAGAAGAAAAAAAAGAAAAGACCCATGCC   1050
F.thunbergii     TCTTATCTTGATGGGTATCAAGAGAATTGGGAATTGGGAAGACTTAAAGAAGAAAAAAAAGAAAAGACCCATGCC   1050
```

```
F.unibracteata   TATTTCCGGTTTGAAAAACCTCTTATGACTTTTTTTTTCGATTATAAACGATGGAATCGTCCATTTAGATATATA  1125
F.przewalskii    TATTTCCGGTTTGAAAAACCTCTTATGACTTTTTTTTTCGATTATAAACGATGGAATCGTCCATTTAGATATATA  1125
F.hupehensis     TATTTCCGGTTTGAAAAACCTCTTATGACTTTTTTTTTCGATTATAAACGATGGAATCGTCCATTTAGATATATA  1125
F.cirrhosa       TATTTCCGGTTTGAAAAACCTCTTATGACTTTTTTTTTCGATTATAAACGATGGAATCGTCCATTTAGATATATA  1125
F.ussuriensis    CATTTCCGGTTTGAAAAACCTCTTATGACTTTTTTTTTCGATTATAAACGATGGAATCGTCCATTTAGATATATA  1125
F.delavayi       TATTTCCGGTTTGAAAAACCTCTTATGACTTTTTTTTTCGATTATAAACGATGGAATCGTCCATTTAGATATATA  1125
F.taipaiensis    TATTTCCGGTTTGAAAAACCTCTTATGACTTTTTTTTTCGATTATAAACGATGGAATCGTCCATTTAGATATATA  1125
F.wabuensis      TATTTCCGGTTTGAAAAACCTCTTATGACTTTTTTTTTCGATTATAAACGATGGAATCGTCCATTTAGATATATA  1125
F.walujewii      CATTTCCGGTTTGAAAAACCTCTTATGACTTTTTTTTTCGATTATAAACGATGGAATCGTCCATTTAGATATATA  1125
F.pallidiflora   CATTTCCGGTTTGAAAAACCTCTTATGACTTTTTTTTTCGATTATAAACGATGGAATCGTCCATTTAGATATATA  1125
F.thunbergii     TATTTCCGGTTTGAAAAACCTCTTATGACTTTTTTTTTCGATTATAAACGATGGAATCGTCCATTTAGATATATA  1125

F.unibracteata   AAAAATGATCTATTTGAAAATGCTGTACGAAATGAAATGTCACAATATTTTTTTTATACATGTCCAAGTGATGGA  1200
F.przewalskii    AAAAATGATCTATTTGAAAATGCTGTACGAAATGAAATGTCACAATATTTTTTTTATACATGTCCAAGTGATGGA  1200
F.hupehensis     AAAAATGATCTATTTGAAAATGCTGTACGAAATGAAATGTCACAATATTTTTTTTATACATGTCCAAGTGATGGA  1200
F.cirrhosa       AAAAATGATCTATTTGAAAATGCTGTACGAAATGAAATGTCACAATATTTTTTTTATACATGTCCAAGTGATGGA  1200
F.ussuriensis    AAAAATGATCTATTTGAAAATGCTGTACGAAATGAAATGTCACAATATTTTTTTTATACATGTCCAAGTGATGGA  1200
F.delavayi       AAAAATGATCTATTTGAAAATGCTGTACGAAATGAAATGTCACAATATTTTTTTTATACATGTCCAAGTGATGGA  1200
F.taipaiensis    AAAAATGATCTATTTGAAAATGCTGTACGAAATGAAATGTCACAATATTTTTTTTATACATGTCCAAGTGATGGA  1200
F.wabuensis      AAAAATGATCTATTTGAAAATGCTGTACGAAATGAAATGTCACAATATTTTTTTTATACATGTCCAAGTGATGGA  1200
F.walujewii      AAAAATGATCTATTTGAAAATGCTGTACGAAATGAAATGTCACAATATTTTTTTTATACATGTCCAAGTGATGGA  1200
F.pallidiflora   AAAAATGATCTATTTGAAAATGCTGTACGAAATGAAATGTCACAATATTTTTTTTATACATGTCCAAGTGATGGA  1200
F.thunbergii     AAAAATGATCTATTTGAAAATGCTGTACGAAATGAAATGTCACAATATTTTTTTTATACATGTCCAAGTGATGGA  1200

F.unibracteata   AAAGAAAAAATATCTTTTACGTATCCGTCAAGTTTATCAACTTTTTCAGAAATGATAGAACGCAAAATTTATTTA  1275
F.przewalskii    AAAGAAAAAATATCTTTTACGTATCCGTCAAGTTTATCAACTTTTTCAGAAATGATAGAACGCAAAATTTCTTTA  1275
F.hupehensis     AAAGAAAAAATATCTTTTACGTATCCGTCAAGTTTATCAACTTTTTCAGAAATGATAGAACGCAAAATTTCTTTA  1275
F.cirrhosa       AAAGAAAAAATATCTTTTACGTATCCGTCAAGTTTATCAACTTTTTCAGAAATGATAGAACGCAAAATTTCTTTA  1275
F.ussuriensis    AAAGAAAAAATCTCTTTTACGTATCCGTCAAGTTTATCAACTTTTTCAGAAATGATAGAACGCAAAATTTCATTA  1275
F.delavayi       AAAGAAAAAATATCTTTTACGTATCCGTCAAGTTTATCAACTTTTTCAGAAATGATAGAACGCAAAATTTCTTTA  1275
F.taipaiensis    AAAGAAAAAATATCTTTTACGTATCCGTCAAGTTTATCAACTTTTTCAGAAATGATAGAACGCAAAATTTCTTTA  1275
F.wabuensis      AAAGAAAAAATATCTTTTACGTATCCGTCAAGTTTATCAACTTTTTCAGAAATGATAGAACGCAAAATTTATTTA  1275
F.walujewii      AAAGAAAAAATATCTTTTACGTATCCGTCAAGTTTATCAACTTTTTCAGAAATGATAGAACGCAAAATTTCTTTA  1275
F.pallidiflora   AAAGCAAAAATATCTTTTACGTATCCGTCAAGTTTATCAACTTTTTCAGAAATGATAGAACGCAAAATTTCTTTA  1275
F.thunbergii     AAAGAAAAAATATCTTTTACGTATCCGTCAAGTTTATCAACTTTTTCAGAAATGATAGAACGCAAAATTTCTTTA  1275

F.unibracteata   TATACAATAAAAAAACTAAGTCATCAAGACTTATATAATTATTGGATTTATACCAATGAACAAAAAAAGTTCAAC  1350
F.przewalskii    TATACAATAAAAAAACTAAGTCATCAAGACTTATATAATTATTGGATTTATACCAATGAACAAAAAAAGTTCAAC  1350
F.hupehensis     TACACAATAAAAAAACTAAGTCATCAAGACTTATATAATTATTGGATTTATACCAATGAACAAAAAAAGTTCAAC  1350
F.cirrhosa       TATACAATAAAAAAACTAAGTCATCAAGACTTATATAATTATTGGATTTATACCAATGAACAAAAAAAGTTCAAC  1350
F.ussuriensis    TACACAATGAAAAACCTAAGTCATCAAAACTTATATAATTATTGGATTTATACCAATGAACAAAAAAAGTTCAAC  1350
F.delavayi       TATACAATAAAAAAACTAAGTCATCAAGACTTATATAATTATTGGATTTATACCAATGAACAAAAAAAGTTCAAC  1350
F.taipaiensis    TATACAATAAAAAAACTAAGTCATCAAGACTTATATAATTATTGGATTTATACCAATGAACAAAAAAAGTTCAAC  1350
F.wabuensis      TATACAATAAAAAAACTAAGTCATCAAGACTTATATAATTATTGGATTTATACCAATGAACAAAAAAAGTTCAAC  1350
F.walujewii      TACACAATAAAAAAACTAAGTAATCAAGACTTATATAATTATTGGATTTATACCAATGAACAAAAAAAGTTCAAC  1350
F.pallidiflora   TACACAATAAAAAAACTAAGTAATCAAGACTTATATAATTATTGGATTTATACCAATGAACAAAAAAAGTTCAAC  1350
F.thunbergii     TACACAATAAAAAAACTAAGTCATCAAGACTTATATAATTATTGGATTTATACCAATGAACAAAAAAAGTTCAAC  1350

F.unibracteata   TTAAGAAATGAATTGATAAGTCGAATAAAAGTTCTAGAAAAAGAAAAGGGATTTATTCTTCTAGACATGCTTGAA  1425
F.przewalskii    TTAAGAAATGAATTGATAAGTCGAATAAAAGTTCTAGAAAAAGAAAAGGGATTTATTCTTCTAGACATGCTTGAA  1425
F.hupehensis     TTAAGAAATGAATTGATAAGTCGAATAAAAGTTCTAGAAAAAGAAAAGGGATTTCTTCTTCTAGACATGCTTGAA  1425
F.cirrhosa       TTAAGAAATGAATTGATAAGTCGAATAAAAGTTCTAGAAAAAGAAAAGGGATTTATTCTTCTAGACATGCTTGAA  1425
F.ussuriensis    TTAAGAAATGAATTGATAAGTCGAATAAAAGTTCTAGAAAAAGAAAAGGGATTTCTTCTTCTAGACATGCTTGAA  1425
F.delavayi       TTAAGAAATGAATTGATAAGTCGAATAAAAGTTCTAGAAAAAGAAAAGGGATTTATTCTTCTAGACATGCTTGAA  1425
F.taipaiensis    TTAAGAAATGAATTGATAAGTCGAATAAAAGTTCTAGAAAAAGAAAAGGGATTGATTCTTCTAGACATGCTTGAA  1425
F.wabuensis      TTAAGAAATGAATTGATAAGTCGAATAAAAGTTCTAGAAAAAGAAAAGGGATTTATTCTTCTAGACATGCTTGAA  1425
F.walujewii      TTAAGAAATGAATTGATAAGTCGAATAAAAGTTCTAGAAAAAGAAAAGGGATTTCTTCTTCTGGACATGCTTGAA  1425
F.pallidiflora   TTAAGAAATGAATTGATAAGTCGAATAAAAGTTCTAGAAAAAGAAAAGGGATTTCTTCTTCTAGACATGCTTGAA  1425
F.thunbergii     TTAAGAAATGAATTGATAAGTCGAATAAAAGTTCTAGAAAAAGAAAAGGGATTTCTTCTTCTAGACATGCTTGAA  1425

F.unibracteata   AAAAGGGCCAGACTATACAATGATGA......AAATGAAAAAGAATGCTTGTCTAAAACGTATGATCCTTTTTTT  1494
F.przewalskii    AAAAGGGCCAGACTATACAATGATGA......AAATGAAAAAGAATGCTTGTCTAAAACGTATGATCCTTTTTTA  1494
F.hupehensis     AAAAGGGCCAGACTATACAATGATGA......AAATGAAAAAGAATGCTTGTCTAAAACCTATGATCCTTTTTTA  1494
F.cirrhosa       AAAAGGGCCAGACTATACAATGATGA......AAATGAAAAAGAATGCTTGTCTAAAACGTATGATCCTTTTTTA  1494
F.ussuriensis    AAAAGGGCCAGACTATACAATGATGAGAATGAAAATGAAAAAGAATGCTTGTCTAAAACGTATGATCCTTTTTTA  1500
F.delavayi       AAAAGGGCCAGACTATACAATGATGA......AAATGAAAAAGAATGCTTGTCTAAAACGTATGATCCTTTTTTA  1494
F.taipaiensis    AAAAGGGCCAGACTATACAATGATGA......AAATGAAAAAGAATGCTTGTCTAAAACGTATGATCCTTTTTTA  1494
F.wabuensis      AAAAGGGCCAGACTATACAATGATGA......AAATGAAAAAGAATGCTTGTCTAAAACGTATGATCCTTTTTTT  1494
F.walujewii      AAAAGGGCCAGACTATACAATGATGA......AAATGAAAAAGAATGCTTGTCTAAAACGTATGATCCTTTTTTG  1494
F.pallidiflora   AAAAGGGCCAGACTATACAATGATGA......AAATGAAAAAGAATGCTTGTCTAAAACGTATGATCCTTTTTTG  1494
F.thunbergii     AAAAGGGCCAGACTATACAATGATGA......AAATGAAAAAGAATGCTTGTCTAAAACCTATGATCCTTTTTTA  1494

F.unibracteata   AATGGACCATATCGTGGAATAATAAAAAAGTTCGATTCACACGCAAATATAAATGATTTAATGACTTCTAGAAAA  1569
F.przewalskii    AATGGACCATATCGTGGAATAATAAAAAAGTTCGATTCACACGCAAATATAAATGATTTAATGACTTCTAGAAAA  1569
F.hupehensis     AATGGACCATATCGTGGAATAATAAAAAAGTTCGATTCACGCGCAAATATAAATGATTTAATGACTTCTAGAAAA  1569
F.cirrhosa       AATGGACCATATCGTGGAATAATAAAAAAGTTCGATTCACACGCAAATATAAATGATTTAATGACTTCTAGAAAA  1569
F.ussuriensis    AATGGGCCATATCGTGGAATAATAAAAAAGTTCGATTCACGCGCAAATATAAATGATTTAATGACTTCTAGAAAA  1575
F.delavayi       AATGGACCATATCGTGGAATAATAAAAAAGTTCGATTCACACGCAAATATAAATGATTTAATGACTTCTAGAAAA  1569
F.taipaiensis    AATGGACCATATCGTGGAATAATAAAAAAGTTCAATTCACACGCAAATATAAAAGATTTAATGACTTCTAGAAAG  1569
F.wabuensis      AATGGACCATATCGTGGAATAATAAAAAAGTTCGATTCACACGCAAATATAAATGATTTAATGACTTCTAGAAAA  1569
F.walujewii      AATGGACCATATCGTGGAATAATAAAAAAGTTCGATTCACGCGCAAATATAAATGATTTAATGACTTCTAGAAAA  1569
F.pallidiflora   AATGGACCATATCGTGGAATAATAAAAAAGTTCGATTCACGCGCAAATATAAATGATTTAATGACTTCTAGAAAA  1569
F.thunbergii     AATGGACCATATCGTGGAATAATAAAAAAGTTCGATTCACGCGCAAATATAAATGATTTAATGACTTCTAGAAAA  1569
```

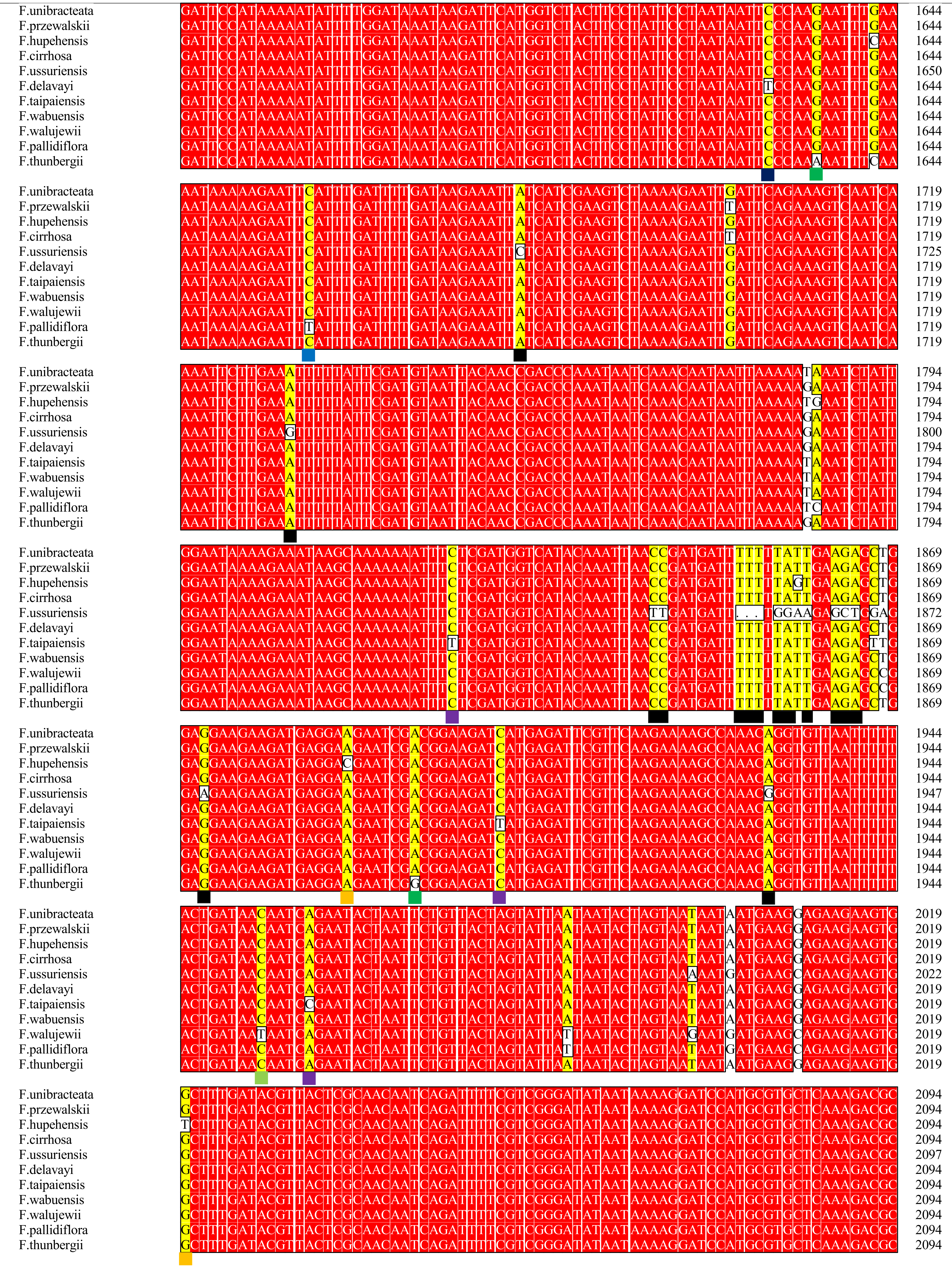
F.unibracteata GATTCCATAAAAATATTTTGGATAAATAAGATTCATGGTCTACTTCCTATTCCTAATAATTCCCAAGAATTTGAA 1644
F.przewalskii GATTCCATAAAAATATTTTGGATAAATAAGATTCATGGTCTACTTCCTATTCCTAATAATTCCCAAGAATTTGAA 1644
F.hupehensis GATTCCATAAAAATATTTTGGATAAATAAGATTCATGGTCTACTTCCTATTCCTAATAATTCCCAAGAATTTCAA 1644
F.cirrhosa GATTCCATAAAAATATTTTGGATAAATAAGATTCATGGTCTACTTCCTATTCCTAATAATTCCCAAGAATTTGAA 1644
F.ussuriensis GATTCCATAAAAATATTTTGGATAAATAAGATTCATGGTCTACTTCCTATTCCTAATAATTCCCAAGAATTTGAA 1650
F.delavayi GATTCCATAAAAATATTTTGGATAAATAAGATTCATGGTCTACTTCCTATTCCTAATAATTTCCAAGAATTTGAA 1644
F.taipaiensis GATTCCATAAAAATATTTTGGATAAATAAGATTCATGGTCTACTTCCTATTCCTAATAATTCCCAAGAATTTGAA 1644
F.wabuensis GATTCCATAAAAATATTTTGGATAAATAAGATTCATGGTCTACTTCCTATTCCTAATAATTCCCAAGAATTTGAA 1644
F.walujewii GATTCCATAAAAATATTTTGGATAAATAAGATTCATGGTCTACTTCCTATTCCTAATAATTCCCAAGAATTTGAA 1644
F.pallidiflora GATTCCATAAAAATATTTTGGATAAATAAGATTCATGGTCTACTTCCTATTCCTAATAATTCCCAAGAATTTGAA 1644
F.thunbergii GATTCCATAAAAATATTTTGGATAAATAAGATTCATGGTCTACTTCCTATTCCTAATAATTCCCAAAAATTTCAA 1644
F.unibracteata AATAAAAAGAATTCATTTGATTTTGATAAGAAATTATCATCGAAGTCTAAAAGAATTGATTCAGAAAGTCAATCA 1719
F.przewalskii AATAAAAAGAATTCATTTGATTTTGATAAGAAATTATCATCGAAGTCTAAAAGAATTTATTCAGAAAGTCAATCA 1719
F.hupehensis AATAAAAAGAATTCATTTGATTTTGATAAGAAATTATCATCGAAGTCTAAAAGAATTGATTCAGAAAGTCAATCA 1719
F.cirrhosa AATAAAAAGAATTCATTTGATTTTGATAAGAAATTATCATCGAAGTCTAAAAGAATTTATTCAGAAAGTCAATCA 1719
F.ussuriensis AATAAAAAGAATTCATTTGATTTTGATAAGAAATTCTCATCGAAGTCTAAAAGAATTGATTCAGAAAGTCAATCA 1725
F.delavayi AATAAAAAGAATTCATTTGATTTTGATAAGAAATTATCATCGAAGTCTAAAAGAATTGATTCAGAAAGTCAATCA 1719
F.taipaiensis AATAAAAAGAATTCATTTGATTTTGATAAGAAATTATCATCGAAGTCTAAAAGAATTGATTCAGAAAGTCAATCA 1719
F.wabuensis AATAAAAAGAATTCATTTGATTTTGATAAGAAATTATCATCGAAGTCTAAAAGAATTGATTCAGAAAGTCAATCA 1719
F.walujewii AATAAAAAGAATTCATTTGATTTTGATAAGAAATTATCATCGAAGTCTAAAAGAATTGATTCAGAAAGTCAATCA 1719
F.pallidiflora AATAAAAAGAATTTATTTGATTTTGATAAGAAATTATCATCGAAGTCTAAAAGAATTGATTCAGAAAGTCAATCA 1719
F.thunbergii AATAAAAAGAATTCATTTGATTTTGATAAGAAATTATCATCGAAGTCTAAAAGAATTGATTCAGAAAGTCAATCA 1719
F.unibracteata AAATTCTTGAAATTTTTATTCGATGTAATTACAACCGACCCAAATAATCAAACAATAATTAAAAATAAATCTATT 1794
F.przewalskii AAATTCTTGAAATTTTTATTCGATGTAATTACAACCGACCCAAATAATCAAACAATAATTAAAAAGAAATCTATT 1794
F.hupehensis AAATTCTTGAAATTTTTATTCGATGTAATTACAACCGACCCAAATAATCAAACAATAATTAAAAATGAATCTATT 1794
F.cirrhosa AAATTCTTGAAATTTTTATTCGATGTAATTACAACCGACCCAAATAATCAAACAATAATTAAAAAGAAATCTATT 1794
F.ussuriensis AAATTCTTGAAGTTTTTATTCGATGTAATTACAACCGACCCAAATAATCAAACAATAATTAAAAAGAAATCTATT 1800
F.delavayi AAATTCTTGAAATTTTTATTCGATGTAATTACAACCGACCCAAATAATCAAACAATAATTAAAAAGAAATCTATT 1794
F.taipaiensis AAATTCTTGAAATTTTTATTCGATGTAATTACAACCGACCCAAATAATCAAACAATAATTAAAAATAAATCTATT 1794
F.wabuensis AAATTCTTGAAATTTTTATTCGATGTAATTACAACCGACCCAAATAATCAAACAATAATTAAAAATAAATCTATT 1794
F.walujewii AAATTCTTGAAATTTTTATTCGATGTAATTACAACCGACCCAAATAATCAAACAATAATTAAAAATAAATCTATT 1794
F.pallidiflora AAATTCTTGAAATTTTTATTCGATGTAATTACAACCGACCCAAATAATCAAACAATAATTAAAAATCAATCTATT 1794
F.thunbergii AAATTCTTGAAATTTTTATTCGATGTAATTACAACCGACCCAAATAATCAAACAATAATTAAAAAGAAATCTATT 1794
F.unibracteata GGAATAAAAGAAATAAGCAAAAAAATTTCTCGATGGTCATACAAATTAACCGATGATTTTTTTATTGAAGAGCTG 1869
F.przewalskii GGAATAAAAGAAATAAGCAAAAAAATTTCTCGATGGTCATACAAATTAACCGATGATTTTTTTATTGAAGAGCTG 1869
F.hupehensis GGAATAAAAGAAATAAGCAAAAAAATTTCTCGATGGTCATACAAATTAACCGATGATTTTTTTAGTGAAGAGCTG 1869
F.cirrhosa GGAATAAAAGAAATAAGCAAAAAAATTTCTCGATGGTCATACAAATTAACCGATGATTTTTTTATTGAAGAGCTG 1869
F.ussuriensis GGAATAAAAGAAATAAGCAAAAAAATTTCTCGATGGTCATACAAATTAATTGATGATT...TGGAAGAGCTGGAG 1872
F.delavayi GGAATAAAAGAAATAAGCAAAAAAATTTCTCGATGGTCATACAAATTAACCGATGATTTTTTTATTGAAGAGCTG 1869
F.taipaiensis GGAATAAAAGAAATAAGCAAAAAAATTTTTCGATGGTCATACAAATTAACCGATGATTTTTTTATTGAAGAGTTG 1869
F.wabuensis GGAATAAAAGAAATAAGCAAAAAAATTTCTCGATGGTCATACAAATTAACCGATGATTTTTTTATTGAAGAGCTG 1869
F.walujewii GGAATAAAAGAAATAAGCAAAAAAATTTCTCGATGGTCATACAAATTAACCGATGATTTTTTTATTGAAGAGCCG 1869
F.pallidiflora GGAATAAAAGAAATAAGCAAAAAAATTTCTCGATGGTCATACAAATTAACCGATGATTTTTTTATTGAAGAGCCG 1869
F.thunbergii GGAATAAAAGAAATAAGCAAAAAAATTTCTCGATGGTCATACAAATTAACCGATGATTTTTTTATTGAAGAGCTG 1869
F.unibracteata GAGGAAGAAGATGAGGAAGAATCGACGGAAGATCATGAGATTCGTTCAAGAAAAGCCAAACAGGTGTTAATTTTT 1944
F.przewalskii GAGGAAGAAGATGAGGAAGAATCGACGGAAGATCATGAGATTCGTTCAAGAAAAGCCAAACAGGTGTTAATTTTT 1944
F.hupehensis GAGGAAGAAGATGAGGACGAATCGACGGAAGATCATGAGATTCGTTCAAGAAAAGCCAAACAGGTGTTAATTTTT 1944
F.cirrhosa GAGGAAGAAGATGAGGAAGAATCGACGGAAGATCATGAGATTCGTTCAAGAAAAGCCAAACAGGTGTTAATTTTT 1944
F.ussuriensis GAAGAAGAAGATGAGGAAGAATCGACGGAAGATCATGAGATTCGTTCAAGAAAAGCCAAACGGGTGTTAATTTTT 1947
F.delavayi GAGGAAGAAGATGAGGAAGAATCGACGGAAGATCATGAGATTCGTTCAAGAAAAGCCAAACAGGTGTTAATTTTT 1944
F.taipaiensis GAGGAAGAAGATGAGGAAGAATCGACGGAAGATTATGAGATTCGTTCAAGAAAAGCCAAACAGGTGTTAATTTTT 1944
F.wabuensis GAGGAAGAAGATGAGGAAGAATCGACGGAAGATCATGAGATTCGTTCAAGAAAAGCCAAACAGGTGTTAATTTTT 1944
F.walujewii GAGGAAGAAGATGAGGAAGAATCGACGGAAGATCATGAGATTCGTTCAAGAAAAGCCAAACAGGTGTTAATTTTT 1944
F.pallidiflora GAGGAAGAAGATGAGGAAGAATCGACGGAAGATCATGAGATTCGTTCAAGAAAAGCCAAACAGGTGTTAATTTTT 1944
F.thunbergii GAGGAAGAAGATGAGGAAGAATCGGCGGAAGATCATGAGATTCGTTCAAGAAAAGCCAAACAGGTGTTAATTTTT 1944
F.unibracteata ACTGATAACAATCAGAATACTAATTCTGTTACTAGTATTAATAATACTAGTAATAATAATGAAGGAGAAGAAGTG 2019
F.przewalskii ACTGATAACAATCAGAATACTAATTCTGTTACTAGTATTAATAATACTAGTAATAATAATGAAGGAGAAGAAGTG 2019
F.hupehensis ACTGATAACAATCAGAATACTAATTCTGTTACTAGTATTAATAATACTAGTAATAATAATGAAGGAGAAGAAGTG 2019
F.cirrhosa ACTGATAACAATCAGAATACTAATTCTGTTACTAGTATTAATAATACTAGTAATAATAATGAAGGAGAAGAAGTG 2019
F.ussuriensis ACTGATAACAATCAGAATACTAATTCTGTTACTAGTATTAATAATACTAGTAAAAATGATGAAGCAGAAGAAGTG 2022
F.delavayi ACTGATAACAATCAGAATACTAATTCTGTTACTAGTATTAATAATACTAGTAATAATAATGAAGGAGAAGAAGTG 2019
F.taipaiensis ACTGATAACAATCCGAATACTAATTCTGTTACTAGTATTAATAATACTAGTAATAATAATGAAGGAGAAGAAGTG 2019
F.wabuensis ACTGATAACAATCAGAATACTAATTCTGTTACTAGTATTAATAATACTAGTAATAATAATGAAGGAGAAGAAGTG 2019
F.walujewii ACTGATAATAATCAGAATACTAATTCTGTTACTAGTATTATTAATACTAGTAAGAATGATGAAGCAGAAGAAGTG 2019
F.pallidiflora ACTGATAACAATCAGAATACTAATTCTGTTACTAGTATTATTAATACTAGTAATAATGATGAAGCAGAAGAAGTG 2019
F.thunbergii ACTGATAACAATCAGAATACTAATTCTGTTACTAGTATTAATAATACTAGTAATAATAATGAAGGAGAAGAAGTG 2019
F.unibracteata GCTTTGATACGTTACTCGCAACAATCAGATTTTCGTCGGGATATAATAAAAGGATCCATGCGTGCTCAAAGACGC 2094
F.przewalskii GCTTTGATACGTTACTCGCAACAATCAGATTTTCGTCGGGATATAATAAAAGGATCCATGCGTGCTCAAAGACGC 2094
F.hupehensis TCTTTGATACGTTACTCGCAACAATCAGATTTTCGTCGGGATATAATAAAAGGATCCATGCGTGCTCAAAGACGC 2094
F.cirrhosa GCTTTGATACGTTACTCGCAACAATCAGATTTTCGTCGGGATATAATAAAAGGATCCATGCGTGCTCAAAGACGC 2094
F.ussuriensis GCTTTGATACGTTACTCGCAACAATCAGATTTTCGTCGGGATATAATAAAAGGATCCATGCGTGCTCAAAGACGC 2097
F.delavayi GCTTTGATACGTTACTCGCAACAATCAGATTTTCGTCGGGATATAATAAAAGGATCCATGCGTGCTCAAAGACGC 2094
F.taipaiensis GCTTTGATACGTTACTCGCAACAATCAGATTTTCGTCGGGATATAATAAAAGGATCCATGCGTGCTCAAAGACGC 2094
F.wabuensis GCTTTGATACGTTACTCGCAACAATCAGATTTTCGTCGGGATATAATAAAAGGATCCATGCGTGCTCAAAGACGC 2094
F.walujewii GCTTTGATACGTTACTCGCAACAATCAGATTTTCGTCGGGATATAATAAAAGGATCCATGCGTGCTCAAAGACGC 2094
F.pallidiflora GCTTTGATACGTTACTCGCAACAATCAGATTTTCGTCGGGATATAATAAAAGGATCCATGCGTGCTCAAAGACGC 2094
F.thunbergii GCTTTGATACGTTACTCGCAACAATCAGATTTTCGTCGGGATATAATAAAAGGATCCATGCGTGCTCAAAGACGC 2094

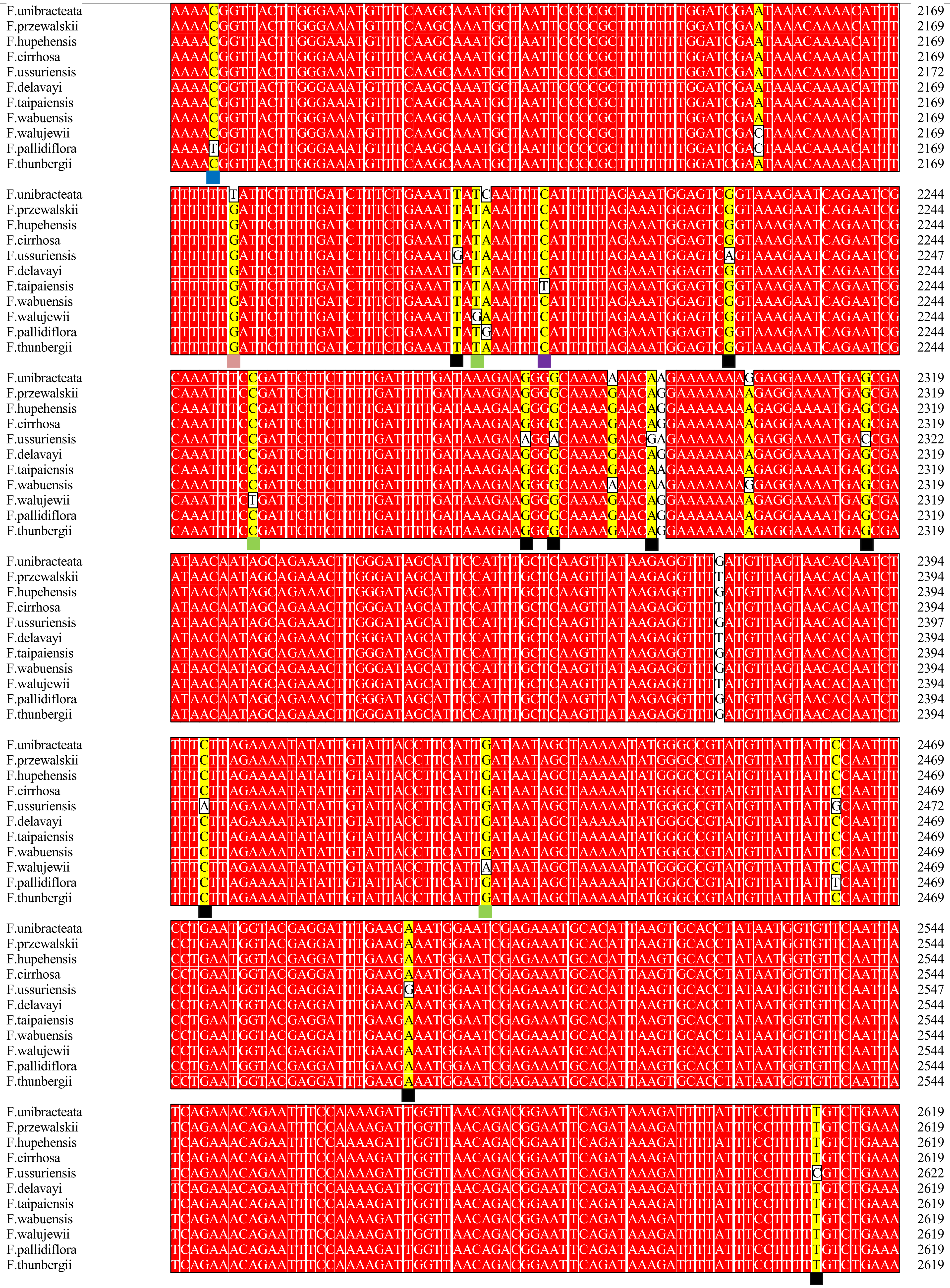
F.unibracteata
F.przewalskii
F.hupehensis
F.cirrhosa
F.ussuriensis
F.delavayi
F.taipaiensis
F.wabuensis
F.walujewii
F.pallidiflora
F.thunbergii
2169
2172
2244
2247
2319
2322
2394
2397
2469
2472
2544
2547
2619
2622

F.unibracteata	CCTTGGCGAAGACAAAGATCTAAGGTACGATCTCATTATATAGATTCAATGAAAAAACAAGTAAAAAAGACACT	2694
F.przewalskii	CCTTGGCGAAAACAAAGATCTAAGGTACGATCTCATTATATAGATTCAATGAAAAAACAAGTAAAAAAGACACT	2694
F.hupehensis	CCTTGGCGAAGACAAAGATCTAAGGTACGATCTCATTATATAGATTCAATGAAAAAACAAGTAAAAAAGACACT	2694
F.cirrhosa	CCTTGGCGAAAACAAAGATCTAAGGTACGATCTCATTATATAGATTCAATGAAAAAACAAGTAAAAAAGACACT	2694
F.ussuriensis	CCTTGGCGAAGACAAAGATCTAAGGTACGATCTCATTATATAGATTCAATGAAAAAACAAGGAAAAAAGACAAT	2697
F.delavayi	CCTTGGCGAAGACAAAGATCTAAGGTACGATCTCATTATATAGATTCAATGAAAAAACAAGTAAAAAAGACACT	2694
F.taipaiensis	CCTTGGCGAAGACAAAGATCTAAGGTACGATCTCATTATATAGATTCAATGAAAAAACAAGTAAAAAAGACACT	2694
F.wabuensis	CCTTGGCGAAGACAAAGATCTAAGGTACGATCTCATTATATAGATTCAATGAAAAAACAAGTAAAAAAGACACT	2694
F.walujewii	CCTTGGCGAAGACAAAGGTCTAAGGTACGATCTCATTATATAGATTCAATGAAAAAACAAGTAAAAAAGACAAT	2694
F.pallidiflora	CCTTGGCGAAGACAAAGATCTAAGGTACGATCTCATTATATAGATTCAATGAAAAAACAAGTAAAAAAGACAAT	2694
F.thunbergii	CCTTGGCGAAGACAAAGATCTAAGGTACGATCTCATTATATAGATTCAATGAAAAAACAAGTAAAAAAGACACT	2694

F.unibracteata	TTTTCTTTTTTAACAGTATGGGGAATGGAAACGGAACTTCCCTTTGGTTCTCCCCGAAAACAATTTGCTTTTTTTT	2769
F.przewalskii	TTTTCTTTTTTAACAGTATGGGGAATGGAAACGGAACTTCCCTTTGGTTCTCCCCGAAAACAATTTTCTTTTTTTT	2769
F.hupehensis	TTTTATTTTTTAACAGTATGGGGAATGGAAACGGAACTTCCCTTTGGTTCTCCCCGAAAACAACTTTCTTTTTTTT	2769
F.cirrhosa	TTTTCTTTTTTAACAGTATGGGGAATGGAAACGGAACTTCCCTTTGGTTCTCCCCGAAAACAATTTTCTTTTTTTT	2769
F.ussuriensis	TTTTCTTTTTTAACAATATGGGGAGTAGAAGTGGAACTTCCCTTTGGTTCTCCCCAAAAACAACTTTCTTTTTTTT	2772
F.delavayi	TTTTCTTTTTTAACAGTATGGGGAATGGAAACGGAACTTCCCTTTGGTTCTCCCCGAAAACAACTTTCTTTTTTTT	2769
F.taipaiensis	TTTTATTTTTTAACAGTATGGGGAATGGAAACGGAACTTCCCTTTGGTTCTCCCCGAAAACAATTTTCTTTTTTTT	2769
F.wabuensis	TTTCATTTTTTAACAGTATGGGGAATGGAAACGGAACTTCCCTTTGGTTCTCCCCGAAAACAATTTGCTTTTTTTT	2769
F.walujewii	TTTTCTTTTTTAACAGTATGGGGAATGGAAACGGAACTTCCCTTTGGTTCTCCCCGAAAACAACTTTCTTTTTTTT	2769
F.pallidiflora	TTTTCTTTTTTAACAGTATGGGGAATGGAAACGGAACTTCCCTTTGGTTCTCCCCGAAAACAACTTTCTTTTTTTT	2769
F.thunbergii	TTTTATTTTTTAACAGTATGGGGAATGGAAACGGAACTTCCCTTTGGTTCTCCCCGAAAACAACTTTCTTTTTTTT	2769

F.unibracteata	GAACCCATTTTTTAAAGAATTCAAAAGAAAAATTAGAAAGCTAAAAAAACAATTTTTTCTCGTTCTAAGGGTCTTA	2844
F.przewalskii	GAACCCATTTTTTAAAGAATTCAAAAGAAAAATTAGAAAGCTAAAAAAACCATTTTTTCTCGTTCTAAGGGTCTTA	2844
F.hupehensis	GAACCCATTTTTTAAAGAATTCAAAAGAAAAATTAGAAAGCTAAAAAAACCATTTTTTCTCGTTCTAAGGGTCTTA	2844
F.cirrhosa	GAACCCATTTTTTAAAGAATTCAAAAGAAAAATTAGAAAGCTAAAAAAACCATTTTTTCTCGTTCTAAGGGTCTTA	2844
F.ussuriensis	GAACCCATTTTTTAAAGAATTCGAAAGAAAAATTAGAAAGCTAAAAAAACTATTTTTTCTCGTTCTAAGGGTCTTA	2847
F.delavayi	GAACCCATTTTTTAAAGAATTCAAAAGAAAAATTAGAAAGCTAAAAAAACCATTTTTTCTCGTTCTAAGGGTCTTA	2844
F.taipaiensis	GAACCCATTTTTTAAAGAATTCAAAATAAAAATTAGAAAGCTAAAAAAACCATTTTTTCTCGTTCTAAGGGTCTTA	2844
F.wabuensis	GAACCCATTTTTTAAAGAATTCAAAAGAAAAATTAGAAAGCTAAAAAAACAATTTTTTCTCGTTCTAAGGGTCTTA	2844
F.walujewii	GAACCCATTTTTTAAAGAATTCAAAAGAAAAATTAGAAAGCTAAAAAAACAATTTTTTCTCGTTCTAAGGGTCTTA	2844
F.pallidiflora	GACCCCATTTTTTAAAGAATTCAAAATAAAAATTAGAAAGCTAAAAAAACAATTTTTTTTCGTTCTAAGGGTCTTA	2844
F.thunbergii	GAACCCATTTTTTAAAGAATTCAAAAGAAAAATTAGAAAGCTAAAAAAACCATTTTTTCTCGTTCTAAGGGTCTTA	2844

F.unibracteata	AAGGAAAGAGGAAAATGGTCTCTACAAATTTTAAAAGAAAAAAAAGAATGGGTCATAAAAACAGCTCTTGTTATA	2919
F.przewalskii	AAGGAAAGAGGAAAATGGTCTCTACAAATTTTAAAAGAAAAAAAAGAATGGGTCATAAAAACAGCTCTTGTTATA	2919
F.hupehensis	AAGGAAAGAGTAAAATGGTCTCTACAAATTTTAAAAGAAAAAAAAGAATGGGTCATAAAAACAGCTCTTGTTATA	2919
F.cirrhosa	AAGGAAAGAGGAAAATGGTCTCTACAAATTTTAAAAGAAAAAAAAGAATGGGTCATAAAAACAGCTCTTGTTATA	2919
F.ussuriensis	AAGGAAAGAGTAAAATGGTCTCTACAAATTTCAAAAGAAAAAAAAGAATGGGTCATCAAAACAGTTCTTGTTATA	2922
F.delavayi	AAGGAAAGAGGAAAATGGTCTCTACAAATTTTAAAAGAAAAAAAAGAATGGGTCATAAAAACAGCTCTTGTTATA	2919
F.taipaiensis	AAGGAAAGAGGAAAATGGTCTTTACAAATTTTAAAAGAAAAAAAAGAATGGGTCATAAAAACAGCTCTTGTTATA	2919
F.wabuensis	AAGGAAAGAGGAAAATGGTCTCTACAAATTTTAAAAGAAAAAAAAGAATGGGTCATAAAAACAGCTCTTGTTATA	2919
F.walujewii	AAGGAAAGAGGAAAATGGTCTCTACAAATTTTAAAAGAAAAAAAAGAATGGGTCATAAAAACAGCTCTTGTTATA	2919
F.pallidiflora	AAGGAAAGAGGAAAATGGTTTCTACAAATTTTAAAAGAAAAAAAAGAATGGGTCATAAAAACAGCTCTTGTTATA	2919
F.thunbergii	AAGGAAAGAGGAAAATGGTCTCTACAAATTTTAAAAGAAAAAAAAGAATGGGTCATAAAAACAGCTCTTGTTATA	2919

F.unibracteata	AAGCGAATAATGAAAGAAAAAGTAAATCCGATTTTTTCATTTGAATTGAAGAAGGTGAAAGTCTATAAACCAAAC	2994
F.przewalskii	AAGCGAATAATGAAAGAAAAAGTAAATCCGATTTTTTCATTTGAATTGAAGAAGGTGAAAGTCTATAAACCAAAC	2994
F.hupehensis	AAGCGAATAATGAAAGAAAAAGTAAATCCGATTTTTTCATTTGAATTGAAGAAGGTGAAAGTCTATAAACCAAAT	2994
F.cirrhosa	AAGCGAATAATGAAAGAAAAAGTAAATCCGATTTTTTCATTTGAATTGAAGAAGGTGAAAGTCTATAAACCAAAC	2994
F.ussuriensis	AAGCGAATAATGAAAGAAAAAGTAAATCTGATTTTTTCATTTGAATTGAAGAAGGTGAAAGTATATAAACCAAAT	2997
F.delavayi	AAGCGAATAATGAAAG......TAAATCCGATTTTTTCATTTGAATTGAAGAAGGTGAAAGTCTATAAACCAAAT	2988
F.taipaiensis	AAGCGAATAATGAAAGAAAAAGTAAATCCGATTTTTTCATTTGAATTGAAGAAGGTGAAAGTCTATAAACCAAAC	2994
F.wabuensis	AAGCGAATAATGAAAGAAAAAGTAAATCCGATTTTTTCATTTGAATTGAAGAAGGTGAAAGTCTATAAACCAAAC	2994
F.walujewii	AAGCGAATAATGAAAGAAAAAGTCAATCCGATTTTTTCATTTGAATTGAAGAAGGTGAAAGTCTATAAACCAAAT	2994
F.pallidiflora	AAGCGAATAATGAAAGAAAAAGTCAATCCGATTTTTTCATTTGAATTGAAGAAGGTGAAAGTCTATAAACCAAAT	2994
F.thunbergii	AAGCGAATAATGAAAGAAAAAGTAAATCCGATTTTTTCATTTGAATTGAAGAAGGTGAAAGTCTATAAACCAAAT	2994

F.unibracteata	AAAAATGGAAAAGATTCAAAAATAAATAATAAAATTAACCATGAAGGGACCATACCAATTCGATCTATGAATTGG	3069
F.przewalskii	AAAAATGGAAAAGATTCAAAAATAAATAATAAAATTAACCATGAAGGGACCATACCAATTCGATCTATGAATTGG	3069
F.hupehensis	AAAAATGGAAAAGATTCAAAAATAAATAATAAAATTAACCATGAAGGGACCATACCAATTAGATCTATGAATTGG	3069
F.cirrhosa	AAAAATGGAAAAGATTCAAAAATAAATAATAAAATTAACCATGAAGGGACCATACCAATTCGATCTATGAATTGG	3069
F.ussuriensis	AAAAATGGAAAAGATTCAAAAATAAATAATAAAATTAACCATGAATGGACCATACCAATTCGATCTATGAATTGG	3072
F.delavayi	AAAAATGGAAAAGATTCAAAAATAAATAATAAAATTAACCATGAAGGGACCATACCAATTCGATCTATGAATTGG	3063
F.taipaiensis	AAAAATGGAAAAGATTCAAAAATAAATAATAAAATTAACCATGAAGGGACCATACCAATTCGATCTATGAATTGG	3069
F.wabuensis	AAAAATGGAAAAGATTCAAAAATAAATAATAAAATTAACCATGAAGGGACCATACCAATTCGATCTATGAATTGG	3069
F.walujewii	AAAAATGGAAAAGATTCAAAAATAAATAAAAAAATTAACCATGAAGGGACCATACCAATTCGATCTATGAATTGG	3069
F.pallidiflora	AAAAATGGAAAAGATTCAAAAATAAATAAAAAAATTAACCATGAAGGGACCATACCAATTCGATCTATGAATTGG	3069
F.thunbergii	AAAAATGGAAAAGATTCAAAAATAAATAATAAAATTAACCATGAAGGGACCATACCAATTCGATCTATGAATTGG	3069

F.unibracteata	ACAAATTATTCACTCATAGAAAAAAAAATGAAAGATCTTTATGATAGGATAATCACAACCAAGATCAAATAGAA	3144
F.przewalskii	ACAAATTATTCACTCATAGAAAAAAAAATGAAAGATCTTTATGATAGGATAATCACAACCAAGATCAAATAGAA	3144
F.hupehensis	ACAAATTATTCACTCATAGAAAAAAAAATGAAAGATCTTTATGATAGGATAATCACAACCAAGATCAAATAGAA	3144
F.cirrhosa	ACAAATTATTCACTCATAGAAAAAAAAATGAAAGATCTTTATGATAGGATAATCACAACCAAGATCAAATAGAA	3144
F.ussuriensis	ACAAATTATTCACTCATAGAAAAAAAAATGAAAGATCTTTATGATAGGATAATCACAACCAAGATCAAATAGAA	3147
F.delavayi	ACAAATTATTCACTCATAGAAAAAAAAATGAAAGATCTTTATGATAGGATAATCACAACCAAGATCAAATAGAA	3138
F.taipaiensis	ACAAATTATTCACTCATAGAAAAAAAAATGAAAGATCTTTATGATAGGATAATCACAACTAAGATCAAATAGAA	3144
F.wabuensis	ACAAATTATTCACTCATAGAAAAAAAAATGAAAGATCTTTATGATAGGATAATCACAACCAAGATCAAATAGAA	3144
F.walujewii	ACAAATTATTCACTCATAGAAAAAAAAATGAAAGATCTTTATGATAGGATAATCACAACCAAGATCAAATAGAA	3144
F.pallidiflora	ACAAATTATTCACTCATAGAAAAAAAAATGAAAGATCTTTATGATAGGATAATCACAACCAAGATCAAATAGAA	3144
F.thunbergii	ACAAATTATTCACTCATAGAAAAAAAAATGAAAGATCTTTATGATAGGATAATCACAACCAAGATCAAATAGAA	3144

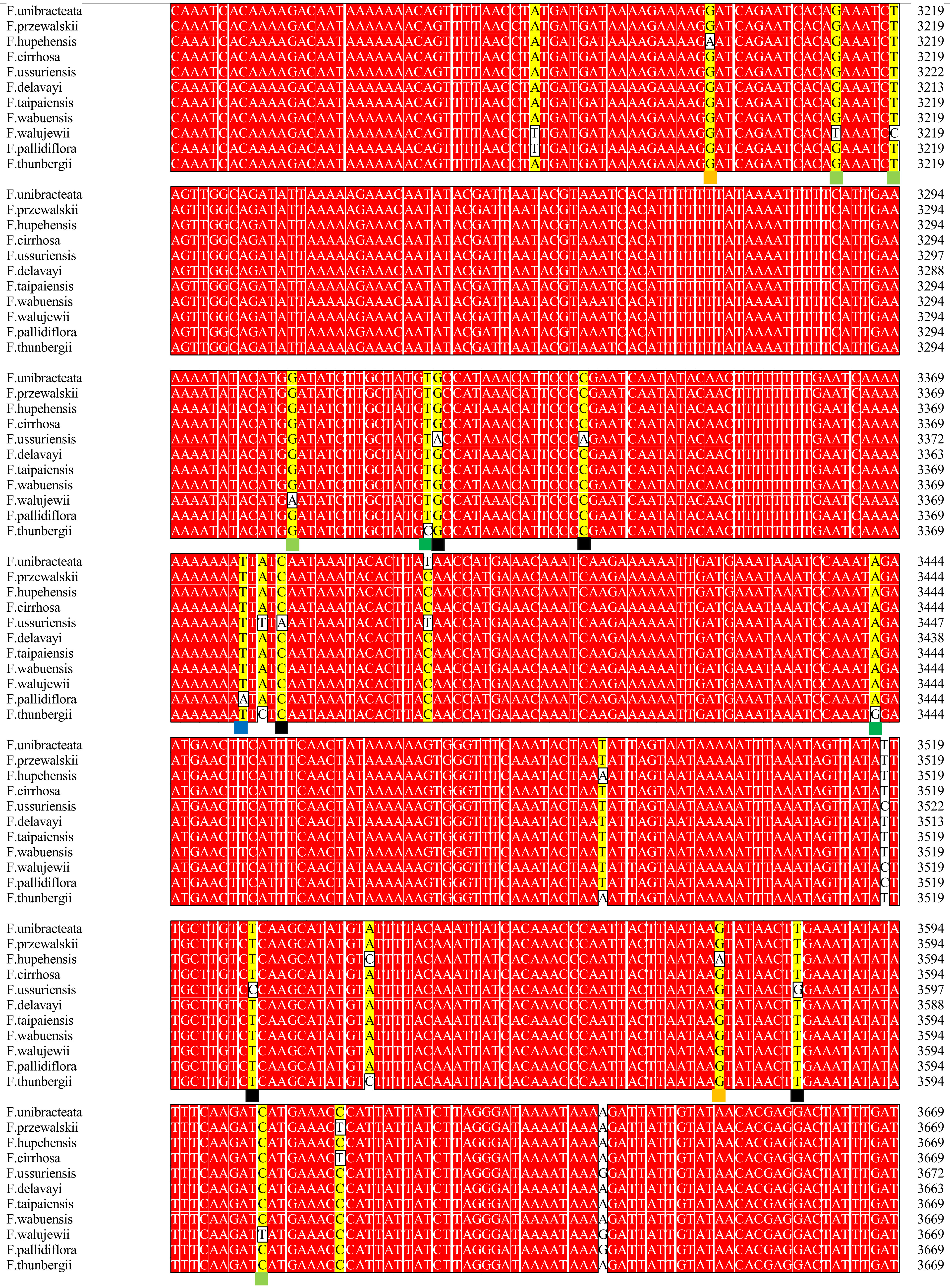
F.unibracteata CAAATCACAAAAGACAATAAAAAAACAGTTTTAACCTATGATGATAAAAGAAAAGGATCAGAATCACAGAAATCT 3219
F.przewalskii CAAATCACAAAAGACAATAAAAAAACAGTTTTAACCTATGATGATAAAAGAAAAGGATCAGAATCACAGAAATCT 3219
F.hupehensis CAAATCACAAAAGACAATAAAAAAACAGTTTTAACCTATGATGATAAAAGAAAAGAATCAGAATCACAGAAATCT 3219
F.cirrhosa CAAATCACAAAAGACAATAAAAAAACAGTTTTAACCTATGATGATAAAAGAAAAGGATCAGAATCACAGAAATCT 3219
F.ussuriensis CAAATCACAAAAGACAATAAAAAAACAGTTTTAACCTATGATGATAAAAGAAAAGGATCAGAATCACAGAAATCT 3222
F.delavayi CAAATCACAAAAGACAATAAAAAAACAGTTTTAACCTATGATGATAAAAGAAAAGGATCAGAATCACAGAAATCT 3213
F.taipaiensis CAAATCACAAAAGACAATAAAAAAACAGTTTTAACCTATGATGATAAAAGAAAAGGATCAGAATCACAGAAATCT 3219
F.wabuensis CAAATCACAAAAGACAATAAAAAAACAGTTTTAACCTATGATGATAAAAGAAAAGGATCAGAATCACAGAAATCT 3219
F.walujewii CAAATCACAAAAGACAATAAAAAAACAGTTTTAACCTTTGATGATAAAAGAAAAGGATCAGAATCACATAAATCC 3219
F.pallidiflora CAAATCACAAAAGACAATAAAAAAACAGTTTTAACCTTTGATGATAAAAGAAAAGGATCAGAATCACAGAAATCT 3219
F.thunbergii CAAATCACAAAAGACAATAAAAAAACAGTTTTAACCTATGATGATAAAAGAAAAGGATCAGAATCACAGAAATCT 3219
F.unibracteata AGTTGGCAGATATTAAAAAGAAACAATATACGATTAATACGTAAATCACATTTTTTTATAAAATTTTTCATTGAA 3294
F.przewalskii AGTTGGCAGATATTAAAAAGAAACAATATACGATTAATACGTAAATCACATTTTTTTATAAAATTTTTCATTGAA 3294
F.hupehensis AGTTGGCAGATATTAAAAAGAAACAATATACGATTAATACGTAAATCACATTTTTTTATAAAATTTTTCATTGAA 3294
F.cirrhosa AGTTGGCAGATATTAAAAAGAAACAATATACGATTAATACGTAAATCACATTTTTTTATAAAATTTTTCATTGAA 3294
F.ussuriensis AGTTGGCAGATATTAAAAAGAAACAATATACGATTAATACGTAAATCACATTTTTTTATAAAATTTTTCATTGAA 3297
F.delavayi AGTTGGCAGATATTAAAAAGAAACAATATACGATTAATACGTAAATCACATTTTTTTATAAAATTTTTCATTGAA 3288
F.taipaiensis AGTTGGCAGATATTAAAAAGAAACAATATACGATTAATACGTAAATCACATTTTTTTATAAAATTTTTCATTGAA 3294
F.wabuensis AGTTGGCAGATATTAAAAAGAAACAATATACGATTAATACGTAAATCACATTTTTTTATAAAATTTTTCATTGAA 3294
F.walujewii AGTTGGCAGATATTAAAAAGAAACAATATACGATTAATACGTAAATCACATTTTTTTATAAAATTTTTCATTGAA 3294
F.pallidiflora AGTTGGCAGATATTAAAAAGAAACAATATACGATTAATACGTAAATCACATTTTTTTATAAAATTTTTCATTGAA 3294
F.thunbergii AGTTGGCAGATATTAAAAAGAAACAATATACGATTAATACGTAAATCACATTTTTTTATAAAATTTTTCATTGAA 3294
F.unibracteata AAAATATACATGGATATCTTGCTATGTGCCATAAACATTCCCCGAATCAATATACAACTTTTTTTTGAATCAAAA 3369
F.przewalskii AAAATATACATGGATATCTTGCTATGTGCCATAAACATTCCCCGAATCAATATACAACTTTTTTTTGAATCAAAA 3369
F.hupehensis AAAATATACATGGATATCTTGCTATGTGCCATAAACATTCCCCGAATCAATATACAACTTTTTTTTGAATCAAAA 3369
F.cirrhosa AAAATATACATGGATATCTTGCTATGTGCCATAAACATTCCCCGAATCAATATACAACTTTTTTTTGAATCAAAA 3369
F.ussuriensis AAAATATACATGGATATCTTGCTATGTACCATAAACATTCCCAGAATCAATATACAACTTTTTTTTGAATCAAAA 3372
F.delavayi AAAATATACATGGATATCTTGCTATGTGCCATAAACATTCCCCGAATCAATATACAACTTTTTTTTGAATCAAAA 3363
F.taipaiensis AAAATATACATGGATATCTTGCTATGTGCCATAAACATTCCCCGAATCAATATACAACTTTTTTTTGAATCAAAA 3369
F.wabuensis AAAATATACATGGATATCTTGCTATGTGCCATAAACATTCCCCGAATCAATATACAACTTTTTTTTGAATCAAAA 3369
F.walujewii AAAATATACATGAATATCTTGCTATGTGCCATAAACATTCCCCGAATCAATATACAACTTTTTTTTGAATCAAAA 3369
F.pallidiflora AAAATATACATGGATATCTTGCTATGTGCCATAAACATTCCCCGAATCAATATACAACTTTTTTTTGAATCAAAA 3369
F.thunbergii AAAATATACATGGATATCTTGCTATGCGCCATAAACATTCCCCGAATCAATATACAACTTTTTTTTGAATCAAAA 3369
F.unibracteata AAAAAAATTATCAATAAATACACTTATAACCATGAACAAATCAAGAAAAAATTGATGAAATAAATCCAAATAGA 3444
F.przewalskii AAAAAAATTATCAATAAATACACTTACAACCATGAACAAATCAAGAAAAAATTGATGAAATAAATCCAAATAGA 3444
F.hupehensis AAAAAAATTATCAATAAATACACTTACAACCATGAACAAATCAAGAAAAAATTGATGAAATAAATCCAAATAGA 3444
F.cirrhosa AAAAAAATTATCAATAAATACACTTACAACCATGAACAAATCAAGAAAAAATTGATGAAATAAATCCAAATAGA 3444
F.ussuriensis AAAAAAATTTTAAATAAATACACTTATAACCATGAACAAATCAAGAAAAAATTGATGAAATAAATCCAAATAGA 3447
F.delavayi AAAAAAATTATCAATAAATACACTTACAACCATGAACAAATCAAGAAAAAATTGATGAAATAAATCCAAATAGA 3438
F.taipaiensis AAAAAAATTATCAATAAATACACTTACAACCATGAACAAATCAAGAAAAAATTGATGAAATAAATCCAAATAGA 3444
F.wabuensis AAAAAAATTATCAATAAATACACTTACAACCATGAACAAATCAAGAAAAAATTGATGAAATAAATCCAAATAGA 3444
F.walujewii AAAAAAATTATCAATAAATACACTTACAACCATGAACAAATCAAGAAAAAATTGATGAAATAAATCCAAATAGA 3444
F.pallidiflora AAAAAAAATATCAATAAATACACTTACAACCATGAACAAATCAAGAAAAAATTGATGAAATAAATCCAAATAGA 3444
F.thunbergii AAAAAAATTCTCAATAAATACACTTACAACCATGAACAAATCAAGAAAAAATTGATGAAATAAATCCAAATGGA 3444
F.unibracteata ATGAACTTCATTTCAACTATAAAAAAGTGGGTTTCAAATACTAATATTAGTAATAAAAATTTAAATAGTTATATT 3519
F.przewalskii ATGAACTTCATTTCAACTATAAAAAAGTGGGTTTCAAATACTAATATTAGTAATAAAAATTTAAATAGTTATATT 3519
F.hupehensis ATGAACTTCATTTCAACTATAAAAAAGTGGGTTTCAAATACTAAAATTAGTAATAAAAATTTAAATAGTTATATT 3519
F.cirrhosa ATGAACTTCATTTCAACTATAAAAAAGTGGGTTTCAAATACTAATATTAGTAATAAAAATTTAAATAGTTATATT 3519
F.ussuriensis ATGAACTTCATTTCAACTATAAAAAAGTGGGTTTCAAATACTAATATTAGTAATAAAAATTTAAATAGTTATACT 3522
F.delavayi ATGAACTTCATTTCAACTATAAAAAAGTGGGTTTCAAATACTAATATTAGTAATAAAAATTTAAATAGTTATATT 3513
F.taipaiensis ATGAACTTCATTTCAACTATAAAAAAGTGGGTTTCAAATACTAATATTAGTAATAAAAATTTAAATAGTTATATT 3519
F.wabuensis ATGAACTTCATTTCAACTATAAAAAAGTGGGTTTCAAATACTAATATTAGTAATAAAAATTTAAATAGTTATATT 3519
F.walujewii ATGAACTTCATTTCAACTATAAAAAAGTGGGTTTCAAATACTAATATTAGTAATAAAAATTTAAATAGTTATACT 3519
F.pallidiflora ATGAACTTCATTTCAACTATAAAAAAGTGGGTTTCAAATACTAATATTAGTAATAAAAATTTAAATAGTTATACT 3519
F.thunbergii ATGAACTTCATTTCAACTATAAAAAAGTGGGTTTCAAATACTAAAATTAGTAATAAAAATTTAAATAGTTATATT 3519
F.unibracteata TGCTTGTCTCAAGCATATGTATTTTACAAATTATCACAAACCCAATTACTTAATAAGTATAACTTTGAAATATATA 3594
F.przewalskii TGCTTGTCTCAAGCATATGTATTTTACAAATTATCACAAACCCAATTACTTAATAAGTATAACTTTGAAATATATA 3594
F.hupehensis TGCTTGTCTCAAGCATATGTCTTTTACAAATTATCACAAACCCAATTACTTAATAAATATAACTTTGAAATATATA 3594
F.cirrhosa TGCTTGTCTCAAGCATATGTATTTTACAAATTATCACAAACCCAATTACTTAATAAGTATAACTTTGAAATATATA 3594
F.ussuriensis TGCTTGTCCCAAGCATATGTATTTTACAAATTATCACAAACCCAATTACTTAATAAGTATAACTGGAAATATATA 3597
F.delavayi TGCTTGTCTCAAGCATATGTATTTTACAAATTATCACAAACCCAATTACTTAATAAGTATAACTTTGAAATATATA 3588
F.taipaiensis TGCTTGTCTCAAGCATATGTATTTTACAAATTATCACAAACCCAATTACTTAATAAGTATAACTTTGAAATATATA 3594
F.wabuensis TGCTTGTCTCAAGCATATGTATTTTACAAATTATCACAAACCCAATTACTTAATAAGTATAACTTTGAAATATATA 3594
F.walujewii TGCTTGTCTCAAGCATATGTATTTTACAAATTATCACAAACCCAATTACTTAATAAGTATAACTTTGAAATATATA 3594
F.pallidiflora TGCTTGTCTCAAGCATATGTATTTTACAAATTATCACAAACCCAATTACTTAATAAGTATAACTTTGAAATATATA 3594
F.thunbergii TGCTTGTCTCAAGCATATGTCTTTTACAAATTATCACAAACCCAATTACTTAATAAGTATAACTTTGAAATATATA 3594
F.unibracteata TTTCAAGATCATGAAACCCATTATTATCTTAGGGATAAAATAAAAGATTATTGTATAACACGAGGACTATTTGAT 3669
F.przewalskii TTTCAAGATCATGAAACTCATTATTATCTTAGGGATAAAATAAAAGATTATTGTATAACACGAGGACTATTTGAT 3669
F.hupehensis TTTCAAGATCATGAAACCCATTATTATCTTAGGGATAAAATAAAAGATTATTGTATAACACGAGGACTATTTGAT 3669
F.cirrhosa TTTCAAGATCATGAAACTCATTATTATCTTAGGGATAAAATAAAAGATTATTGTATAACACGAGGACTATTTGAT 3669
F.ussuriensis TTTCAAGATCATGAAACCCATTATTATCTTAGGGATAAAATAAAGGATTATTGTATAACACGAGGACTATTTGAT 3672
F.delavayi TTTCAAGATCATGAAACCCATTATTATCTTAGGGATAAAATAAAAGATTATTGTATAACACGAGGACTATTTGAT 3663
F.taipaiensis TTTCAAGATCATGAAACCCATTATTATCTTAGGGATAAAATAAAAGATTATTGTATAACACGAGGACTATTTGAT 3669
F.wabuensis TTTCAAGATCATGAAACCCATTATTATCTTAGGGATAAAATAAAAGATTATTGTATAACACGAGGACTATTTGAT 3669
F.walujewii TTTCAAGATTATGAAACCCATTATTATCTTAGGGATAAAATAAAGGATTATTGTATAACACGAGGACTATTTGAT 3669
F.pallidiflora TTTCAAGATCATGAAACCCATTATTATCTTAGGGATAAAATAAAGGATTATTGTATAACACGAGGACTATTTGAT 3669
F.thunbergii TTTCAAGATCATGAAACCCATTATTATCTTAGGGATAAAATAAAAGATTATTGTATAACACGAGGACTATTTGAT 3669

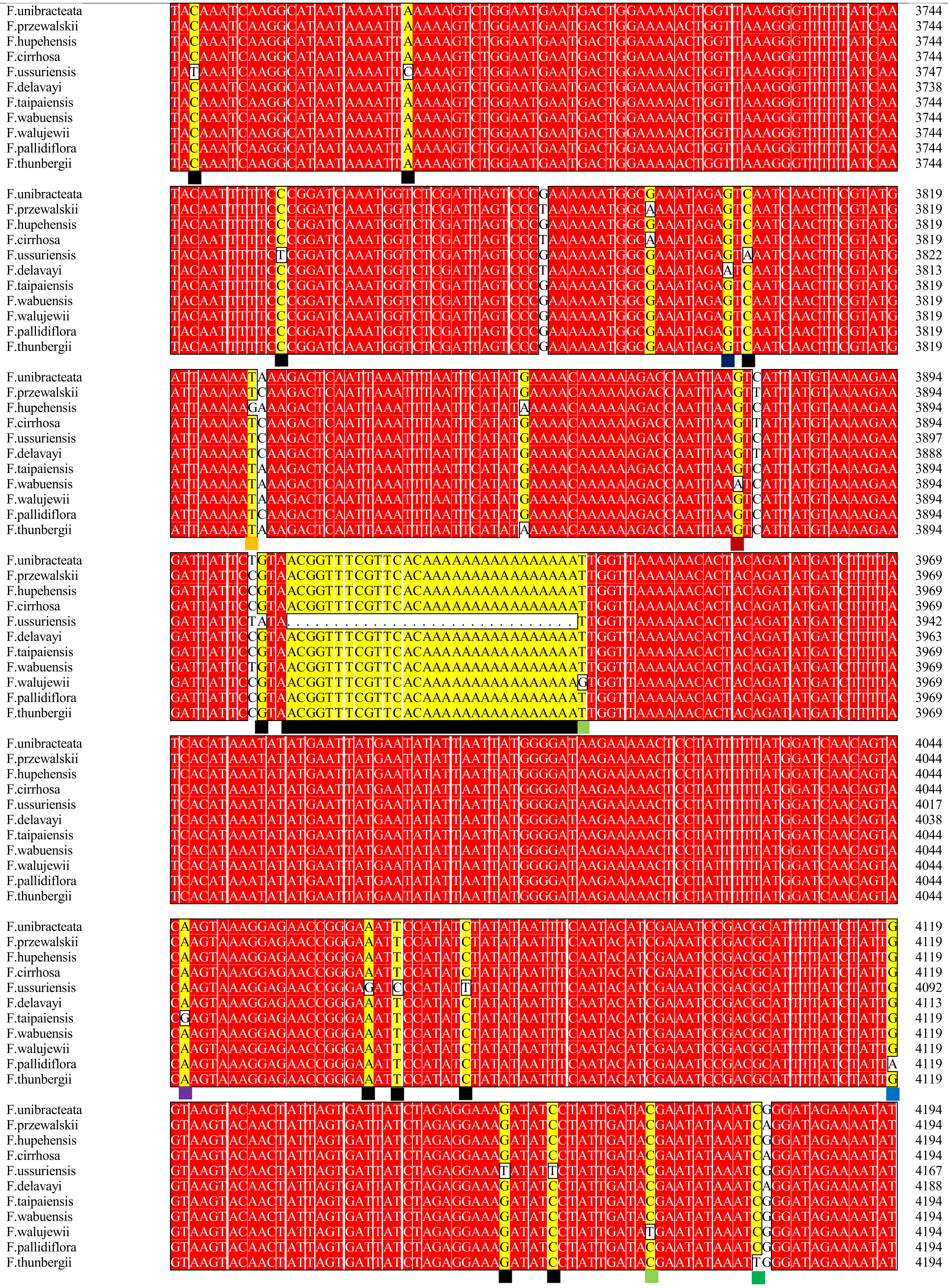
F.unibracteata 3744
F.przewalskii 3744
F.hupehensis 3744
F.cirrhosa 3744
F.ussuriensis 3747
F.delavayi 3738
F.taipaiensis 3744
F.wabuensis 3744
F.walujewii 3744
F.pallidiflora 3744
F.thunbergii 3744
F.unibracteata 3819
F.przewalskii 3819
F.hupehensis 3819
F.cirrhosa 3819
F.ussuriensis 3822
F.delavayi 3813
F.taipaiensis 3819
F.wabuensis 3819
F.walujewii 3819
F.pallidiflora 3819
F.thunbergii 3819
F.unibracteata 3894
F.przewalskii 3894
F.hupehensis 3894
F.cirrhosa 3894
F.ussuriensis 3897
F.delavayi 3888
F.taipaiensis 3894
F.wabuensis 3894
F.walujewii 3894
F.pallidiflora 3894
F.thunbergii 3894
F.unibracteata 3969
F.przewalskii 3969
F.hupehensis 3969
F.cirrhosa 3969
F.ussuriensis 3942
F.delavayi 3963
F.taipaiensis 3969
F.wabuensis 3969
F.walujewii 3969
F.pallidiflora 3969
F.thunbergii 3969
F.unibracteata 4044
F.przewalskii 4044
F.hupehensis 4044
F.cirrhosa 4044
F.ussuriensis 4017
F.delavayi 4038
F.taipaiensis 4044
F.wabuensis 4044
F.walujewii 4044
F.pallidiflora 4044
F.thunbergii 4044
F.unibracteata 4119
F.przewalskii 4119
F.hupehensis 4119
F.cirrhosa 4119
F.ussuriensis 4092
F.delavayi 4113
F.taipaiensis 4119
F.wabuensis 4119
F.walujewii 4119
F.pallidiflora 4119
F.thunbergii 4119
F.unibracteata 4194
F.przewalskii 4194
F.hupehensis 4194
F.cirrhosa 4194
F.ussuriensis 4167
F.delavayi 4188
F.taipaiensis 4194
F.wabuensis 4194
F.walujewii 4194
F.pallidiflora 4194
F.thunbergii 4194

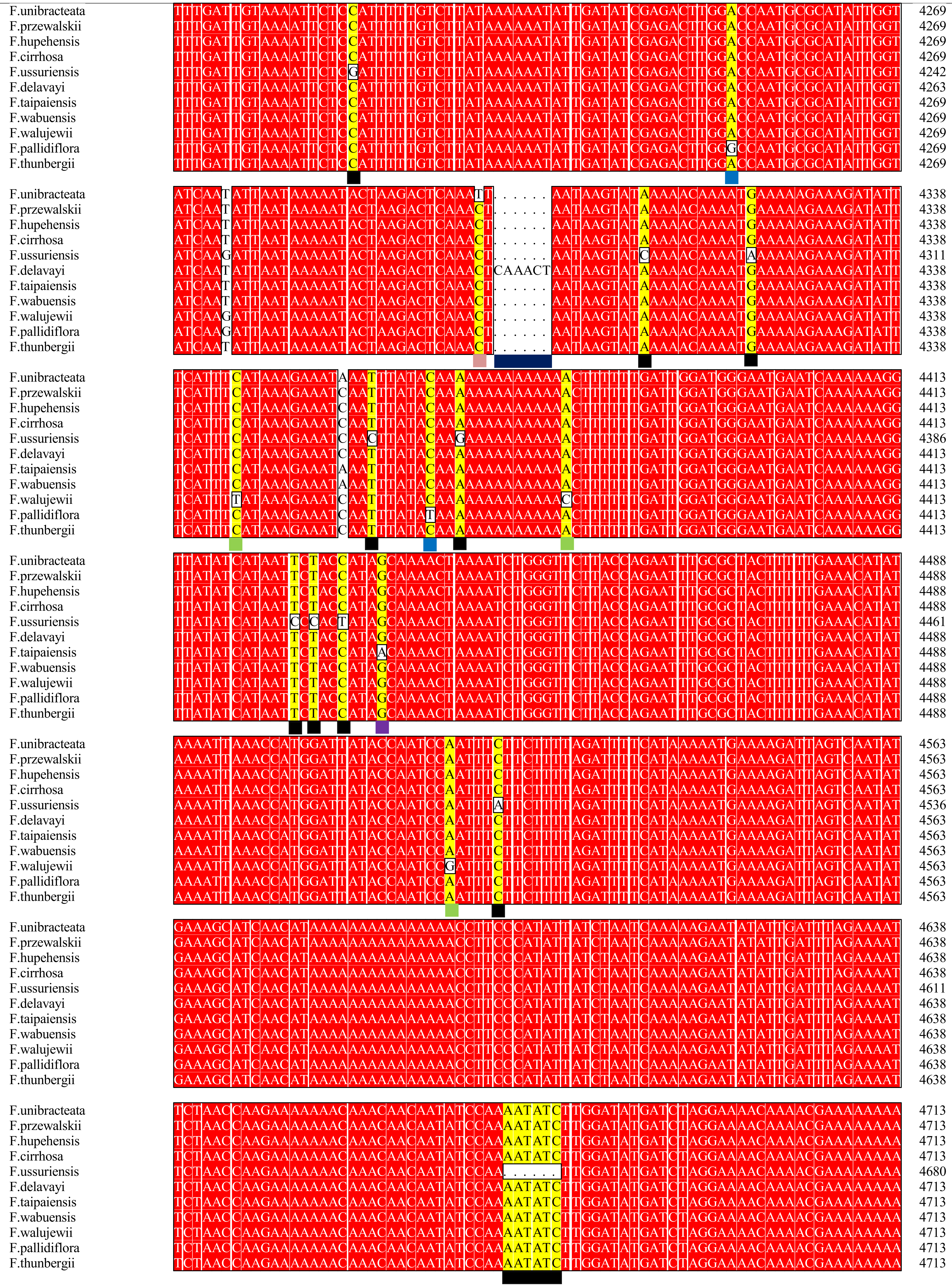
F.unibracteata TTTGATTGTAAAATTCTCCATTTTTGTCTTATAAAAAATATTGATATCGAGACTTGGACCAATGCGCATATTGGT 4269
F.przewalskii TTTGATTGTAAAATTCTCCATTTTTGTCTTATAAAAAATATTGATATCGAGACTTGGACCAATGCGCATATTGGT 4269
F.hupehensis TTTGATTGTAAAATTCTCCATTTTTGTCTTATAAAAAATATTGATATCGAGACTTGGACCAATGCGCATATTGGT 4269
F.cirrhosa TTTGATTGTAAAATTCTCCATTTTTGTCTTATAAAAAATATTGATATCGAGACTTGGACCAATGCGCATATTGGT 4269
F.ussuriensis TTTGATTGTAAAATTCTCGATTTTTGTCTTATAAAAAATATTGATATCGAGACTTGGACCAATGCGCATATTGGT 4242
F.delavayi TTTGATTGTAAAATTCTCCATTTTTGTCTTATAAAAAATATTGATATCGAGACTTGGACCAATGCGCATATTGGT 4263
F.taipaiensis TTTGATTGTAAAATTCTCCATTTTTGTCTTATAAAAAATATTGATATCGAGACTTGGACCAATGCGCATATTGGT 4269
F.wabuensis TTTGATTGTAAAATTCTCCATTTTTGTCTTATAAAAAATATTGATATCGAGACTTGGACCAATGCGCATATTGGT 4269
F.walujewii TTTGATTGTAAAATTCTCCATTTTTGTCTTATAAAAAATATTGATATCGAGACTTGGACCAATGCGCATATTGGT 4269
F.pallidiflora TTTGATTGTAAAATTCTCCATTTTTGTCTTATAAAAAATATTGATATCGAGACTTGGGCCAATGCGCATATTGGT 4269
F.thunbergii TTTGATTGTAAAATTCTCCATTTTTGTCTTATAAAAAATATTGATATCGAGACTTGGACCAATGCGCATATTGGT 4269
F.unibracteata ATCAATATTAATAAAAATACTAAGACTCAAATT......AATAAGTATAAAAACAAAATGAAAAAGAAAGATATT 4338
F.przewalskii ATCAATATTAATAAAAATACTAAGACTCAAACT......AATAAGTATAAAAACAAAATGAAAAAGAAAGATATT 4338
F.hupehensis ATCAATATTAATAAAAATACTAAGACTCAAACT......AATAAGTATAAAAACAAAATGAAAAAGAAAGATATT 4338
F.cirrhosa ATCAATATTAATAAAAATACTAAGACTCAAACT......AATAAGTATAAAAACAAAATGAAAAAGAAAGATATT 4338
F.ussuriensis ATCAAGATTAATAAAAATACTAAGACTCAAACT......AATAAGTATCAAAACAAAATAAAAAAGAAAGATATT 4311
F.delavayi ATCAATATTAATAAAAATACTAAGACTCAAACTCAAACTAATAAGTATAAAAACAAAATGAAAAAGAAAGATATT 4338
F.taipaiensis ATCAATATTAATAAAAATACTAAGACTCAAACT......AATAAGTATAAAAACAAAATGAAAAAGAAAGATATT 4338
F.wabuensis ATCAATATTAATAAAAATACTAAGACTCAAACT......AATAAGTATAAAAACAAAATGAAAAAGAAAGATATT 4338
F.walujewii ATCAAGATTAATAAAAATACTAAGACTCAAACT......AATAAGTATAAAAACAAAATGAAAAAGAAAGATATT 4338
F.pallidiflora ATCAAGATTAATAAAAATACTAAGACTCAAACT......AATAAGTATAAAAACAAAATGAAAAAGAAAGATATT 4338
F.thunbergii ATCAATATTAATAAAAATACTAAGACTCAAACT......AATAAGTATAAAAACAAAATGAAAAAGAAAGATATT 4338
F.unibracteata TCATTTCATAAAGAAATAAATTTATACAAAAAAAAAAAAAACTTTTTTGATTGGATGGGAATGAATCAAAAAAGG 4413
F.przewalskii TCATTTCATAAAGAAATCAATTTATACAAAAAAAAAAAAAACTTTTTTGATTGGATGGGAATGAATCAAAAAAGG 4413
F.hupehensis TCATTTCATAAAGAAATCAATTTATACAAAAAAAAAAAAAACTTTTTTGATTGGATGGGAATGAATCAAAAAAGG 4413
F.cirrhosa TCATTTCATAAAGAAATCAATTTATACAAAAAAAAAAAAAACTTTTTTGATTGGATGGGAATGAATCAAAAAAGG 4413
F.ussuriensis TCATTTCATAAAGAAATCAACTTATACAAGAAAAAAAAAAAACTTTTTTGATTGGATGGGAATGAATCAAAAAAGG 4386
F.delavayi TCATTTCATAAAGAAATCAATTTATACAAAAAAAAAAAAAACTTTTTTGATTGGATGGGAATGAATCAAAAAAGG 4413
F.taipaiensis TCATTTCATAAAGAAATAAATTTATACAAAAAAAAAAAAAACTTTTTTGATTGGATGGGAATGAATCAAAAAAGG 4413
F.wabuensis TCATTTCATAAAGAAATAAATTTATACAAAAAAAAAAAAAACTTTTTTGATTGGATGGGAATGAATCAAAAAAGG 4413
F.walujewii TCATTTTATAAAGAAATCAATTTATACAAAAAAAAAAAAACCTTTTTTGATTGGATGGGAATGAATCAAAAAAGG 4413
F.pallidiflora TCATTTCATAAAGAAATCAATTTATATAAAAAAAAAAAAAACTTTTTTGATTGGATGGGAATGAATCAAAAAAGG 4413
F.thunbergii TCATTTCATAAAGAAATCAATTTATACAAAAAAAAAAAAAACTTTTTTGATTGGATGGGAATGAATCAAAAAAGG 4413
F.unibracteata TTATATCATAATTCTACCATAGCAAAACTAAAATCTGGGTTCTTACCAGAATTTGCGCTACTTTTTGAAACATAT 4488
F.przewalskii TTATATCATAATTCTACCATAGCAAAACTAAAATCTGGGTTCTTACCAGAATTTGCGCTACTTTTTGAAACATAT 4488
F.hupehensis TTATATCATAATTCTACCATAGCAAAACTAAAATCTGGGTTCTTACCAGAATTTGCGCTACTTTTTGAAACATAT 4488
F.cirrhosa TTATATCATAATTCTACCATAGCAAAACTAAAATCTGGGTTCTTACCAGAATTTGCGCTACTTTTTGAAACATAT 4488
F.ussuriensis TTATATCATAATCCCACTATAGCAAAACTAAAATCTGGGTTCTTACCAGAATTTGCGCTACTTTTTGAAACATAT 4461
F.delavayi TTATATCATAATTCTACCATAGCAAAACTAAAATCTGGGTTCTTACCAGAATTTGCGCTACTTTTTGAAACATAT 4488
F.taipaiensis TTATATCATAATTCTACCATAACAAAACTAAAATCTGGGTTCTTACCAGAATTTGCGCTACTTTTTGAAACATAT 4488
F.wabuensis TTATATCATAATTCTACCATAGCAAAACTAAAATCTGGGTTCTTACCAGAATTTGCGCTACTTTTTGAAACATAT 4488
F.walujewii TTATATCATAATTCTACCATAGCAAAACTAAAATCTGGGTTCTTACCAGAATTTGCGCTACTTTTTGAAACATAT 4488
F.pallidiflora TTATATCATAATTCTACCATAGCAAAACTAAAATCTGGGTTCTTACCAGAATTTGCGCTACTTTTTGAAACATAT 4488
F.thunbergii TTATATCATAATTCTACCATAGCAAAACTAAAATCTGGGTTCTTACCAGAATTTGCGCTACTTTTTGAAACATAT 4488
F.unibracteata AAAATTAAACCATGGATTATACCAATCCAATTTCTTCTTTTAGATTTTCATAAAAATGAAAAGATTAGTCAATAT 4563
F.przewalskii AAAATTAAACCATGGATTATACCAATCCAATTTCTTCTTTTAGATTTTCATAAAAATGAAAAGATTAGTCAATAT 4563
F.hupehensis AAAATTAAACCATGGATTATACCAATCCAATTTCTTCTTTTAGATTTTCATAAAAATGAAAAGATTAGTCAATAT 4563
F.cirrhosa AAAATTAAACCATGGATTATACCAATCCAATTTCTTCTTTTAGATTTTCATAAAAATGAAAAGATTAGTCAATAT 4563
F.ussuriensis AAAATTAAACCATGGATTATACCAATCCAATTTATTCTTTTAGATTTTCATAAAAATGAAAAGATTAGTCAATAT 4536
F.delavayi AAAATTAAACCATGGATTATACCAATCCAATTTCTTCTTTTAGATTTTCATAAAAATGAAAAGATTAGTCAATAT 4563
F.taipaiensis AAAATTAAACCATGGATTATACCAATCCAATTTCTTCTTTTAGATTTTCATAAAAATGAAAAGATTAGTCAATAT 4563
F.wabuensis AAAATTAAACCATGGATTATACCAATCCAATTTCTTCTTTTAGATTTTCATAAAAATGAAAAGATTAGTCAATAT 4563
F.walujewii AAAATTAAACCATGGATTATACCAATCCGATTTCTTCTTTTAGATTTTCATAAAAATGAAAAGATTAGTCAATAT 4563
F.pallidiflora AAAATTAAACCATGGATTATACCAATCCAATTTCTTCTTTTAGATTTTCATAAAAATGAAAAGATTAGTCAATAT 4563
F.thunbergii AAAATTAAACCATGGATTATACCAATCCAATTTCTTCTTTTAGATTTTCATAAAAATGAAAAGATTAGTCAATAT 4563
F.unibracteata GAAAGCATCAACATAAAAAAAAAAAAAAAACCTTCCCATATTATCTAATCAAAAAGAATATATTGATTTAGAAAAT 4638
F.przewalskii GAAAGCATCAACATAAAAAAAAAAAAAAAACCTTCCCATATTATCTAATCAAAAAGAATATATTGATTTAGAAAAT 4638
F.hupehensis GAAAGCATCAACATAAAAAAAAAAAAAAAACCTTCCCATATTATCTAATCAAAAAGAATATATTGATTTAGAAAAT 4638
F.cirrhosa GAAAGCATCAACATAAAAAAAAAAAAAAAACCTTCCCATATTATCTAATCAAAAAGAATATATTGATTTAGAAAAT 4638
F.ussuriensis GAAAGCATCAACATAAAAAAAAAAAAAAAACCTTCCCATATTATCTAATCAAAAAGAATATATTGATTTAGAAAAT 4611
F.delavayi GAAAGCATCAACATAAAAAAAAAAAAAAAACCTTCCCATATTATCTAATCAAAAAGAATATATTGATTTAGAAAAT 4638
F.taipaiensis GAAAGCATCAACATAAAAAAAAAAAAAAAACCTTCCCATATTATCTAATCAAAAAGAATATATTGATTTAGAAAAT 4638
F.wabuensis GAAAGCATCAACATAAAAAAAAAAAAAAAACCTTCCCATATTATCTAATCAAAAAGAATATATTGATTTAGAAAAT 4638
F.walujewii GAAAGCATCAACATAAAAAAAAAAAAAAAACCTTCCCATATTATCTAATCAAAAAGAATATATTGATTTAGAAAAT 4638
F.pallidiflora GAAAGCATCAACATAAAAAAAAAAAAAAAACCTTCCCATATTATCTAATCAAAAAGAATATATTGATTTAGAAAAT 4638
F.thunbergii GAAAGCATCAACATAAAAAAAAAAAAAAAACCTTCCCATATTATCTAATCAAAAAGAATATATTGATTTAGAAAAT 4638
F.unibracteata TCTAACCAAGAAAAAAACAAACAACAATATCCAAAATATCTTGGATATGATCTAGGAAAACAAAACGAAAAAAAA 4713
F.przewalskii TCTAACCAAGAAAAAAACAAACAACAATATCCAAAATATCTTGGATATGATCTAGGAAAACAAAACGAAAAAAAA 4713
F.hupehensis TCTAACCAAGAAAAAAACAAACAACAATATCCAAAATATCTTGGATATGATCTAGGAAAACAAAACGAAAAAAAA 4713
F.cirrhosa TCTAACCAAGAAAAAAACAAACAACAATATCCAAAATATCTTGGATATGATCTAGGAAAACAAAACGAAAAAAAA 4713
F.ussuriensis TCTAACCAAGAAAAAAACAAACAACAATATCCAA......TTGGATATGATCTAGGAAAACAAAACGAAAAAAAA 4680
F.delavayi TCTAACCAAGAAAAAAACAAACAACAATATCCAAAATATCTTGGATATGATCTAGGAAAACAAAACGAAAAAAAA 4713
F.taipaiensis TCTAACCAAGAAAAAAACAAACAACAATATCCAAAATATCTTGGATATGATCTAGGAAAACAAAACGAAAAAAAA 4713
F.wabuensis TCTAACCAAGAAAAAAACAAACAACAATATCCAAAATATCTTGGATATGATCTAGGAAAACAAAACGAAAAAAAA 4713
F.walujewii TCTAACCAAGAAAAAAACAAACAACAATATCCAAAATATCTTGGATATGATCTAGGAAAACAAAACGAAAAAAAA 4713
F.pallidiflora TCTAACCAAGAAAAAAACAAACAACAATATCCAAAATATCTTGGATATGATCTAGGAAAACAAAACGAAAAAAAA 4713
F.thunbergii TCTAACCAAGAAAAAAACAAACAACAATATCCAAAATATCTTGGATATGATCTAGGAAAACAAAACGAAAAAAAA 4713

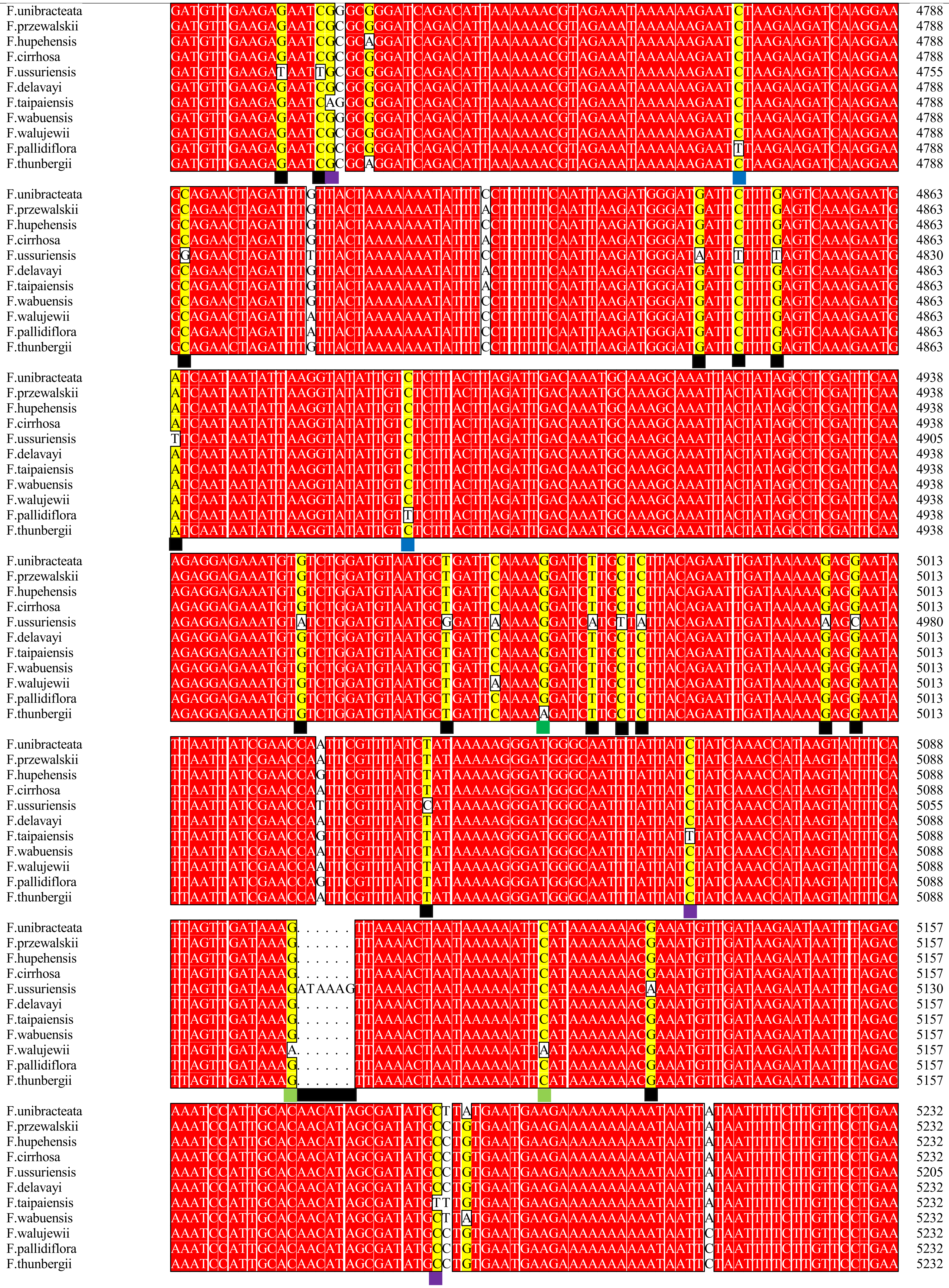

F.unibracteata GATGTTGAAGAGAATCGGGCGGGATCAGACATTAAAAACGTAGAAATAAAAAAGAATCTAAGAGAGATCAAGGAA 4788
F.przewalskii GATGTTGAAGAGAATCGCGCGGGATCAGACATTAAAAACGTAGAAATAAAAAAGAATCTAAGAGAGATCAAGGAA 4788
F.hupehensis GATGTTGAAGAGAATCGCGCAGGATCAGACATTAAAAACGTAGAAATAAAAAAGAATCTAAGAGAGATCAAGGAA 4788
F.cirrhosa GATGTTGAAGAGAATCGCGCGGGATCAGACATTAAAAACGTAGAAATAAAAAAGAATCTAAGAGAGATCAAGGAA 4788
F.ussuriensis GATGTTGAAGATAATTGCGCGGGATCAGACATTAAAAACGTAGAAATAAAAAAGAATCTAAGAGAGATCAAGGAA 4755
F.delavayi GATGTTGAAGAGAATCGCGCGGGATCAGACATTAAAAACGTAGAAATAAAAAAGAATCTAAGAGAGATCAAGGAA 4788
F.taipaiensis GATGTTGAAGAGAATCAGGCGGGATCAGACATTAAAAACGTAGAAATAAAAAAGAATCTAAGAGAGATCAAGGAA 4788
F.wabuensis GATGTTGAAGAGAATCGGGCGGGATCAGACATTAAAAACGTAGAAATAAAAAAGAATCTAAGAGAGATCAAGGAA 4788
F.walujewii GATGTTGAAGAGAATCGCGCGGGATCAGACATTAAAAACGTAGAAATAAAAAAGAATCTAAGAGAGATCAAGGAA 4788
F.pallidiflora GATGTTGAAGAGAATCGCGCGGGATCAGACATTAAAAACGTAGAAATAAAAAAGAATTTAAGAGAGATCAAGGAA 4788
F.thunbergii GATGTTGAAGAGAATCGCGCAGGATCAGACATTAAAAACGTAGAAATAAAAAAGAATCTAAGAGAGATCAAGGAA 4788
F.unibracteata GCAGAACTAGATTTGTTACTAAAAAAATATTTCCTTTTTCAATTAAGATGGGATGATTCTTTGAGTCAAAGAATG 4863
F.przewalskii GCAGAACTAGATTTGTTACTAAAAAAATATTTACTTTTTCAATTAAGATGGGATGATTCTTTGAGTCAAAGAATG 4863
F.hupehensis GCAGAACTAGATTTGTTACTAAAAAAATATTTCCTTTTTCAATTAAGATGGGATGATTCTTTGAGTCAAAGAATG 4863
F.cirrhosa GCAGAACTAGATTTGTTACTAAAAAAATATTTACTTTTTCAATTAAGATGGGATGATTCTTTGAGTCAAAGAATG 4863
F.ussuriensis GGAGAACTAGATTTTTTACTAAAAAAATATTTCCTTTTTCAATTAAGATGGGATAATTTTTTTAGTCAAAGAATG 4830
F.delavayi GCAGAACTAGATTTGTTACTAAAAAAATATTTACTTTTTCAATTAAGATGGGATGATTCTTTGAGTCAAAGAATG 4863
F.taipaiensis GCAGAACTAGATTTGTTACTAAAAAAATATTTACTTTTTCAATTAAGATGGGATGATTCTTTGAGTCAAAGAATG 4863
F.wabuensis GCAGAACTAGATTTGTTACTAAAAAAATATTTCCTTTTTCAATTAAGATGGGATGATTCTTTGAGTCAAAGAATG 4863
F.walujewii GCAGAACTAGATTTATTACTAAAAAAATATTTCCTTTTTCAATTAAGATGGGATGATTCTTTGAGTCAAAGAATG 4863
F.pallidiflora GCAGAACTAGATTTATTACTAAAAAAATATTTCCTTTTTCAATTAAGATGGGATGATTCTTTGAGTCAAAGAATG 4863
F.thunbergii GCAGAACTAGATTTGTTACTAAAAAAATATTTCCTTTTTCAATTAAGATGGGATGATTCTTTGAGTCAAAGAATG 4863
F.unibracteata ATCAATAATATTAAGGTATATTGTCTCTTACTTAGATTGACAAATGCAAAGCAAATTACTATAGCCTCGATTCAA 4938
F.przewalskii ATCAATAATATTAAGGTATATTGTCTCTTACTTAGATTGACAAATGCAAAGCAAATTACTATAGCCTCGATTCAA 4938
F.hupehensis ATCAATAATATTAAGGTATATTGTCTCTTACTTAGATTGACAAATGCAAAGCAAATTACTATAGCCTCGATTCAA 4938
F.cirrhosa ATCAATAATATTAAGGTATATTGTCTCTTACTTAGATTGACAAATGCAAAGCAAATTACTATAGCCTCGATTCAA 4938
F.ussuriensis TTCAATAATATTAAGGTATATTGTCTCTTACTTAGATTGACAAATGCAAAGCAAATTACTATAGCCTCGATTCAA 4905
F.delavayi ATCAATAATATTAAGGTATATTGTCTCTTACTTAGATTGACAAATGCAAAGCAAATTACTATAGCCTCGATTCAA 4938
F.taipaiensis ATCAATAATATTAAGGTATATTGTCTCTTACTTAGATTGACAAATGCAAAGCAAATTACTATAGCCTCGATTCAA 4938
F.wabuensis ATCAATAATATTAAGGTATATTGTCTCTTACTTAGATTGACAAATGCAAAGCAAATTACTATAGCCTCGATTCAA 4938
F.walujewii ATCAATAATATTAAGGTATATTGTCTCTTACTTAGATTGACAAATGCAAAGCAAATTACTATAGCCTCGATTCAA 4938
F.pallidiflora ATCAATAATATTAAGGTATATTGTTTCTTACTTAGATTGACAAATGCAAAGCAAATTACTATAGCCTCGATTCAA 4938
F.thunbergii ATCAATAATATTAAGGTATATTGTCTCTTACTTAGATTGACAAATGCAAAGCAAATTACTATAGCCTCGATTCAA 4938
F.unibracteata AGAGGAGAAATGTGTCTGGATGTAATGCTGATTCAAAAGGATCTTGCTCTTACAGAATTGATAAAAAGAGGAATA 5013
F.przewalskii AGAGGAGAAATGTGTCTGGATGTAATGCTGATTCAAAAGGATCTTGCTCTTACAGAATTGATAAAAAGAGGAATA 5013
F.hupehensis AGAGGAGAAATGTGTCTGGATGTAATGCTGATTCAAAAGGATCTTGCTCTTACAGAATTGATAAAAAGAGGAATA 5013
F.cirrhosa AGAGGAGAAATGTGTCTGGATGTAATGCTGATTCAAAAGGATCTTGCTCTTACAGAATTGATAAAAAGAGGAATA 5013
F.ussuriensis AGAGGAGAAATGTATCTGGATGTAATGCGGATTAAAAAGGATCATGTTATTACAGAATTGATAAAAAAAGCAATA 4980
F.delavayi AGAGGAGAAATGTGTCTGGATGTAATGCTGATTCAAAAGGATCTTGCTCTTACAGAATTGATAAAAAGAGGAATA 5013
F.taipaiensis AGAGGAGAAATGTGTCTGGATGTAATGCTGATTCAAAAGGATCTTGCTCTTACAGAATTGATAAAAAGAGGAATA 5013
F.wabuensis AGAGGAGAAATGTGTCTGGATGTAATGCTGATTCAAAAGGATCTTGCTCTTACAGAATTGATAAAAAGAGGAATA 5013
F.walujewii AGAGGAGAAATGTGTCTGGATGTAATGCTGATTAAAAAGGATCTTGCTCTTACAGAATTGATAAAAAGAGGAATA 5013
F.pallidiflora AGAGGAGAAATGTGTCTGGATGTAATGCTGATTCAAAAGGATCTTGCTCTTACAGAATTGATAAAAAGAGGAATA 5013
F.thunbergii AGAGGAGAAATGTGTCTGGATGTAATGCTGATTCAAAAAGATCTTGCTCTTACAGAATTGATAAAAAGAGGAATA 5013
F.unibracteata TTAATTATCGAACCAATTCGTTTATCTATAAAAAGGGATGGGCAATTTATTATCTATCAAACCATAAGTATTTCA 5088
F.przewalskii TTAATTATCGAACCAATTCGTTTATCTATAAAAAGGGATGGGCAATTTATTATCTATCAAACCATAAGTATTTCA 5088
F.hupehensis TTAATTATCGAACCAGTTCGTTTATCTATAAAAAGGGATGGGCAATTTATTATCTATCAAACCATAAGTATTTCA 5088
F.cirrhosa TTAATTATCGAACCAATTCGTTTATCTATAAAAAGGGATGGGCAATTTATTATCTATCAAACCATAAGTATTTCA 5088
F.ussuriensis TTAATTATCGAACCATTTCGTTTATCCATAAAAAGGGATGGGCAATTTATTATCTATCAAACCATAAGTATTTCA 5055
F.delavayi TTAATTATCGAACCAATTCGTTTATCTATAAAAAGGGATGGGCAATTTATTATCTATCAAACCATAAGTATTTCA 5088
F.taipaiensis TTAATTATCGAACCAGTTCGTTTATCTATAAAAAGGGATGGGCAATTTATTATTTATCAAACCATAAGTATTTCA 5088
F.wabuensis TTAATTATCGAACCAATTCGTTTATCTATAAAAAGGGATGGGCAATTTATTATCTATCAAACCATAAGTATTTCA 5088
F.walujewii TTAATTATCGAACCAATTCGTTTATCTATAAAAAGGGATGGGCAATTTATTATCTATCAAACCATAAGTATTTCA 5088
F.pallidiflora TTAATTATCGAACCAGTTCGTTTATCTATAAAAAGGGATGGGCAATTTATTATCTATCAAACCATAAGTATTTCA 5088
F.thunbergii TTAATTATCGAACCAATTCGTTTATCTATAAAAAGGGATGGGCAATTTATTATCTATCAAACCATAAGTATTTCA 5088
F.unibracteata TTAGTTGATAAAG......TTAAACTAATAAAAAATTCATAAAAAAACGAAATGTTGATAAGAATAATTTAGAC 5157
F.przewalskii TTAGTTGATAAAG......TTAAACTAATAAAAAATTCATAAAAAAACGAAATGTTGATAAGAATAATTTAGAC 5157
F.hupehensis TTAGTTGATAAAG......TTAAACTAATAAAAAATTCATAAAAAAACGAAATGTTGATAAGAATAATTTAGAC 5157
F.cirrhosa TTAGTTGATAAAG......TTAAACTAATAAAAAATTCATAAAAAAACGAAATGTTGATAAGAATAATTTAGAC 5157
F.ussuriensis TTAGTTGATAAAGATAAAGTTAAACTAATAAAAAATTCATAAAAAAACAAAATGTTGATAAGAATAATTTAGAC 5130
F.delavayi TTAGTTGATAAAG......TTAAACTAATAAAAAATTCATAAAAAAACGAAATGTTGATAAGAATAATTTAGAC 5157
F.taipaiensis TTAGTTGATAAAG......TTAAACTAATAAAAAATTCATAAAAAAACGAAATGTTGATAAGAATAATTTAGAC 5157
F.wabuensis TTAGTTGATAAAG......TTAAACTAATAAAAAATTCATAAAAAAACGAAATGTTGATAAGAATAATTTAGAC 5157
F.walujewii TTAGTTGATAAAA......TTAAACTAATAAAAAATTAATAAAAAAACGAAATGTTGATAAGAATAATTTAGAC 5157
F.pallidiflora TTAGTTGATAAAG......TTAAACTAATAAAAAATTCATAAAAAAACGAAATGTTGATAAGAATAATTTAGAC 5157
F.thunbergii TTAGTTGATAAAG......TTAAACTAATAAAAAATTCATAAAAAAACGAAATGTTGATAAGAATAATTTAGAC 5157
F.unibracteata AAATCCATTGCACAACATAGCGATATGCTTATGAATGAAGAAAAAAAAATAATTAATAATTTTCTTGTTCCTGAA 5232
F.przewalskii AAATCCATTGCACAACATAGCGATATGCCTGTGAATGAAGAAAAAAAAATAATTAATAATTTTCTTGTTCCTGAA 5232
F.hupehensis AAATCCATTGCACAACATAGCGATATGCCTGTGAATGAAGAAAAAAAAATAATTAATAATTTTCTTGTTCCTGAA 5232
F.cirrhosa AAATCCATTGCACAACATAGCGATATGCCTGTGAATGAAGAAAAAAAAATAATTAATAATTTTCTTGTTCCTGAA 5232
F.ussuriensis AAATCCATTGCACAACATAGCGATATGCCTGTGAATGAAGAAAAAAAAATAATTAATAATTTTCTTGTTCCTGAA 5205
F.delavayi AAATCCATTGCACAACATAGCGATATGCCTGTGAATGAAGAAAAAAAAATAATTAATAATTTTCTTGTTCCTGAA 5232
F.taipaiensis AAATCCATTGCACAACATAGCGATATGTTTGTGAATGAAGAAAAAAAAATAATTAATAATTTTCTTGTTCCTGAA 5232
F.wabuensis AAATCCATTGCACAACATAGCGATATGCTTATGAATGAAGAAAAAAAAATAATTAATAATTTTCTTGTTCCTGAA 5232
F.walujewii AAATCCATTGCACAACATAGCGATATGCCTGTGAATGAAGAAAAAAAAATAATTCAATAATTTTCTTGTTCCTGAA 5232
F.pallidiflora AAATCCATTGCACAACATAGCGATATGCCTGTGAATGAAGAAAAAAAAATAATTCAATAATTTTCTTGTTCCTGAA 5232
F.thunbergii AAATCCATTGCACAACATAGCGATATGCCTGTGAATGAAGAAAAAAAAATAATTCAATAATTTTCTTGTTCCTGAA 5232

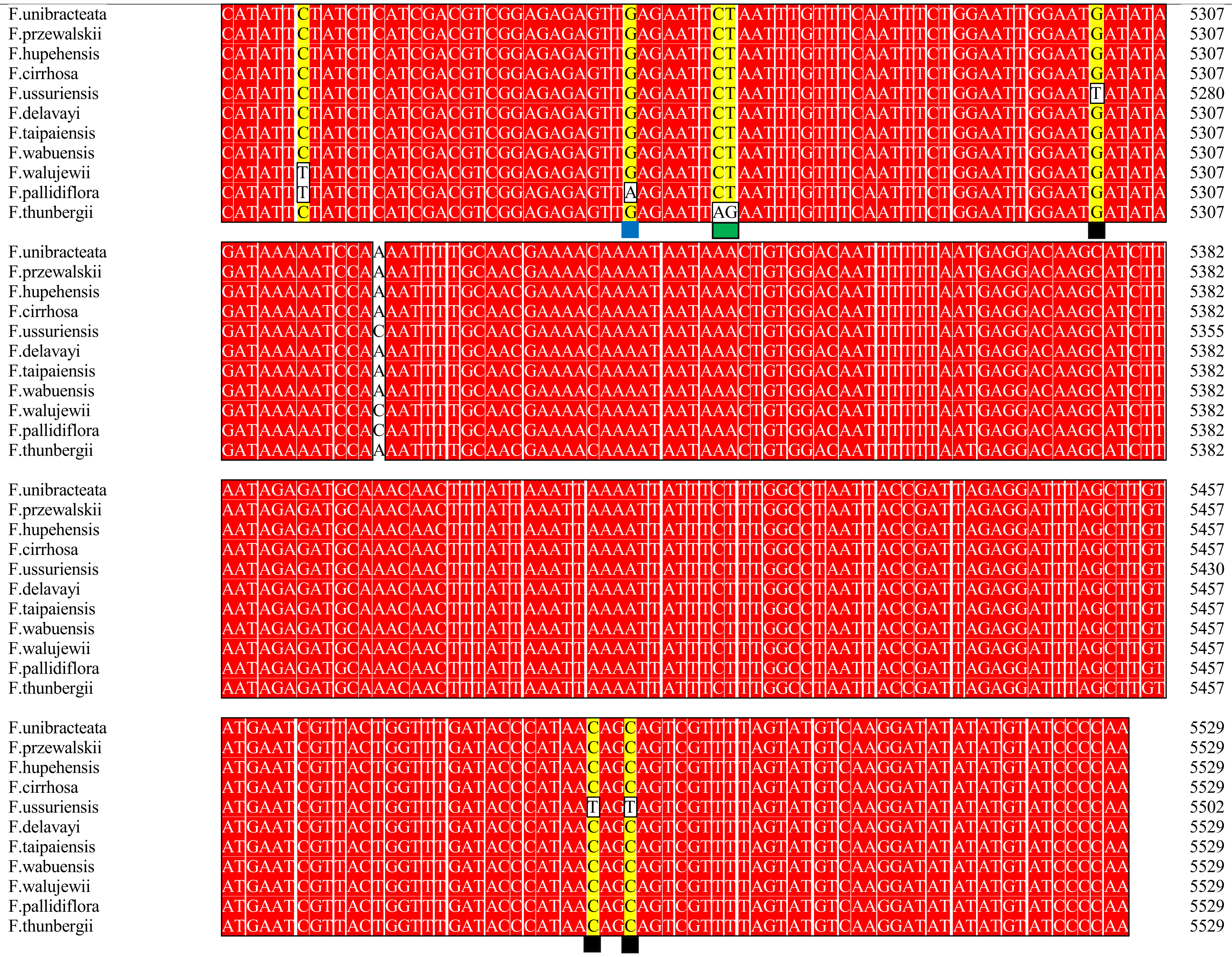

F.unibracteata CATATTCTATCTCATCGACGTCGGAGAGAGTTGAGAATTCTAATTTGTTTCAATTTCTGGAATTGGAATGATATA 5307
F.przewalskii CATATTCTATCTCATCGACGTCGGAGAGAGTTGAGAATTCTAATTTGTTTCAATTTCTGGAATTGGAATGATATA 5307
F.hupehensis CATATTCTATCTCATCGACGTCGGAGAGAGTTGAGAATTCTAATTTGTTTCAATTTCTGGAATTGGAATGATATA 5307
F.cirrhosa CATATTCTATCTCATCGACGTCGGAGAGAGTTGAGAATTCTAATTTGTTTCAATTTCTGGAATTGGAATGATATA 5307
F.ussuriensis CATATTCTATCTCATCGACGTCGGAGAGAGTTGAGAATTCTAATTTGTTTCAATTTCTGGAATTGGAATTATATA 5280
F.delavayi CATATTCTATCTCATCGACGTCGGAGAGAGTTGAGAATTCTAATTTGTTTCAATTTCTGGAATTGGAATGATATA 5307
F.taipaiensis CATATTCTATCTCATCGACGTCGGAGAGAGTTGAGAATTCTAATTTGTTTCAATTTCTGGAATTGGAATGATATA 5307
F.wabuensis CATATTCTATCTCATCGACGTCGGAGAGAGTTGAGAATTCTAATTTGTTTCAATTTCTGGAATTGGAATGATATA 5307
F.walujewii CATATTTTATCTCATCGACGTCGGAGAGAGTTGAGAATTCTAATTTGTTTCAATTTCTGGAATTGGAATGATATA 5307
F.pallidiflora CATATTTTATCTCATCGACGTCGGAGAGAGTTAAGAATTCTAATTTGTTTCAATTTCTGGAATTGGAATGATATA 5307
F.thunbergii CATATTCTATCTCATCGACGTCGGAGAGAGTTGAGAATTAGAATTTGTTTCAATTTCTGGAATTGGAATGATATA 5307
F.unibracteata GATAAAAATCCAAAATTTTGCAACGAAAACAAAATAATAAACTGTGGACAATTTTTAATGAGGACAAGCATCTT 5382
F.przewalskii GATAAAAATCCAAAATTTTGCAACGAAAACAAAATAATAAACTGTGGACAATTTTTAATGAGGACAAGCATCTT 5382
F.hupehensis GATAAAAATCCAAAATTTTGCAACGAAAACAAAATAATAAACTGTGGACAATTTTTAATGAGGACAAGCATCTT 5382
F.cirrhosa GATAAAAATCCAAAATTTTGCAACGAAAACAAAATAATAAACTGTGGACAATTTTTAATGAGGACAAGCATCTT 5382
F.ussuriensis GATAAAAATCCACAATTTTGCAACGAAAACAAAATAATAAACTGTGGACAATTTTTAATGAGGACAAGCATCTT 5355
F.delavayi GATAAAAATCCAAAATTTTGCAACGAAAACAAAATAATAAACTGTGGACAATTTTTAATGAGGACAAGCATCTT 5382
F.taipaiensis GATAAAAATCCAAAATTTTGCAACGAAAACAAAATAATAAACTGTGGACAATTTTTAATGAGGACAAGCATCTT 5382
F.wabuensis GATAAAAATCCAAAATTTTGCAACGAAAACAAAATAATAAACTGTGGACAATTTTTAATGAGGACAAGCATCTT 5382
F.walujewii GATAAAAATCCACAATTTTGCAACGAAAACAAAATAATAAACTGTGGACAATTTTTAATGAGGACAAGCATCTT 5382
F.pallidiflora GATAAAAATCCACAATTTTGCAACGAAAACAAAATAATAAACTGTGGACAATTTTTAATGAGGACAAGCATCTT 5382
F.thunbergii GATAAAAATCCAAAATTTTGCAACGAAAACAAAATAATAAACTGTGGACAATTTTTAATGAGGACAAGCATCTT 5382
F.unibracteata AATAGAGATGCAAACAACTTTATTAAATTAAAATTATTTCTTTGGCCTAATTACCGATTAGAGGATTTAGCTTGT 5457
F.przewalskii AATAGAGATGCAAACAACTTTATTAAATTAAAATTATTTCTTTGGCCTAATTACCGATTAGAGGATTTAGCTTGT 5457
F.hupehensis AATAGAGATGCAAACAACTTTATTAAATTAAAATTATTTCTTTGGCCTAATTACCGATTAGAGGATTTAGCTTGT 5457
F.cirrhosa AATAGAGATGCAAACAACTTTATTAAATTAAAATTATTTCTTTGGCCTAATTACCGATTAGAGGATTTAGCTTGT 5457
F.ussuriensis AATAGAGATGCAAACAACTTTATTAAATTAAAATTATTTCTTTGGCCTAATTACCGATTAGAGGATTTAGCTTGT 5430
F.delavayi AATAGAGATGCAAACAACTTTATTAAATTAAAATTATTTCTTTGGCCTAATTACCGATTAGAGGATTTAGCTTGT 5457
F.taipaiensis AATAGAGATGCAAACAACTTTATTAAATTAAAATTATTTCTTTGGCCTAATTACCGATTAGAGGATTTAGCTTGT 5457
F.wabuensis AATAGAGATGCAAACAACTTTATTAAATTAAAATTATTTCTTTGGCCTAATTACCGATTAGAGGATTTAGCTTGT 5457
F.walujewii AATAGAGATGCAAACAACTTTATTAAATTAAAATTATTTCTTTGGCCTAATTACCGATTAGAGGATTTAGCTTGT 5457
F.pallidiflora AATAGAGATGCAAACAACTTTATTAAATTAAAATTATTTCTTTGGCCTAATTACCGATTAGAGGATTTAGCTTGT 5457
F.thunbergii AATAGAGATGCAAACAACTTTATTAAATTAAAATTATTTCTTTGGCCTAATTACCGATTAGAGGATTTAGCTTGT 5457
F.unibracteata ATGAATCGTTACTGGTTTGATACCCATAACAGCAGTCGTTTTAGTATGTCAAGGATATATATGTATCCCCAA 5529
F.przewalskii ATGAATCGTTACTGGTTTGATACCCATAACAGCAGTCGTTTTAGTATGTCAAGGATATATATGTATCCCCAA 5529
F.hupehensis ATGAATCGTTACTGGTTTGATACCCATAACAGCAGTCGTTTTAGTATGTCAAGGATATATATGTATCCCCAA 5529
F.cirrhosa ATGAATCGTTACTGGTTTGATACCCATAACAGCAGTCGTTTTAGTATGTCAAGGATATATATGTATCCCCAA 5529
F.ussuriensis ATGAATCGTTACTGGTTTGATACCCATAATAGTAGTCGTTTTAGTATGTCAAGGATATATATGTATCCCCAA 5502
F.delavayi ATGAATCGTTACTGGTTTGATACCCATAACAGCAGTCGTTTTAGTATGTCAAGGATATATATGTATCCCCAA 5529
F.taipaiensis ATGAATCGTTACTGGTTTGATACCCATAACAGCAGTCGTTTTAGTATGTCAAGGATATATATGTATCCCCAA 5529
F.wabuensis ATGAATCGTTACTGGTTTGATACCCATAACAGCAGTCGTTTTAGTATGTCAAGGATATATATGTATCCCCAA 5529
F.walujewii ATGAATCGTTACTGGTTTGATACCCATAACAGCAGTCGTTTTAGTATGTCAAGGATATATATGTATCCCCAA 5529
F.pallidiflora ATGAATCGTTACTGGTTTGATACCCATAACAGCAGTCGTTTTAGTATGTCAAGGATATATATGTATCCCCAA 5529
F.thunbergii ATGAATCGTTACTGGTTTGATACCCATAACAGCAGTCGTTTTAGTATGTCAAGGATATATATGTATCCCCAA 5529
