# Supplementary material for: Identification of evolutionary relationships and DNA markers in the medicinally important genus Fritillaria based on chloroplast genomics
Source: PeerJ. 2021 Dec 16;9:e12612. doi: 10.7717/peerj.12612 (PMC8684722; doi:10.7717/peerj.12612)
Supplement: Supplemental Information 7 [file peerj-09-12612-s007.docx]

Table S1 Summary statistics for the assembly of four *Fritillaria* species chloroplast genomes.

| **Genome features** | ***F.unibracteata*** | ***F.przewalskii*** | ***F.delavayi*** | ***F.sinica*** |
| --- | --- | --- | --- | --- |
| Genome size (bp) | 151,076 | 152,043 | 151,940 | 152,016 |
| LSC size (bp) | 81,383 | 81,804 | 81,661 | 81,768 |
| SSC size (bp) | 17,537 | 17,539 | 17,569 | 17,546 |
| IR size (bp) | 26,078 | 26,350 | 26,355 | 26,351 |
| Number of genes | 132 (109) | 133 (110) | 133 (110) | 133 (110) |
| Protein genes [unique] | 86 (77) | 87 (78) | 87 (78) | 87 (78) |
| tRNA genes [unique] | 38 (28) | 38 (28) | 38 (28) | 38 (28) |
| rRNA genes [unique] | 8 (4) | 8 (4) | 8 (4) | 8 (4) |
| Duplicated genes in IR | 39 | 39 | 39 | 39 |
| GC content (%) | 36.96 | 36.94 | 36.96 | 36.95 |
| GC content in LSC (%) | 34.79 | 34.77 | 34.80 | 34.79 |
| GC content in SSC (%) | 30.42 | 30.44 | 30.39 | 30.45 |
| GC content in IR (%) | 42.55 | 42.46 | 42.49 | 42.47 |
| Total reads | 23,755,399 | 26,831,529 | 25,258,295 | 26,585,105 |
| Aligned paired-end reads | 546,756 | 652,632 | 511,467 | 471,385 |
| Assembled reads | 149,891 | 150,858 | 150,755 | 150,831 |
| Average organelle coverage | 1081.3173 | 1291.608 | 1013.4826 | 933.6238 |
| Average insert size (bp) | 322.99 | 331.55 | 341.68 | 336.64 |

Table S2 Information on 15 intron-containing genes in the chloroplast genome of *F.unibracteata*.

| **Gene** | **Location** | **Exon I (bp)** | **Intron I (bp)** | **Exon II (bp）** | **Intron II (bp)** | **Exon III (bp)** |
| --- | --- | --- | --- | --- | --- | --- |
| *ycf3* | LSC | 124 | 740 | 230 | 709 | 159 |
| *clpP* | LSC | 71 | 771 | 294 | 588 | 159 |
| *tRNA-UUU* | LSC | 38 | 2558 | 36 |  |  |
| *tRNA-CGA* | LSC | 32 | 666 | 60 |  |  |
| *atpF* | LSC | 160 | 769 | 410 |  |  |
| *rpoC1* | LSC | 432 | 777 | 1623 |  |  |
| *tRNA-UAA* | LSC | 35 | 533 | 50 |  |  |
| *tRNA-AAU* | LSC | 34 | 585 | 60 |  |  |
| *rpl2* | IRA | 394 | 672 | 428 |  |  |
| *ndhB* | IRA | 775 | 648 | 758 |  |  |
| *tRNA-GAU* | IRA | 36 | 916 | 57 |  |  |
| *tRNA-UGC* | IRA | 37 | 810 | 36 |  |  |
| *ndhA* | SSC | 553 | 1037 | 539 |  |  |
| *tRNA-UGC* | IRB | 38 | 808 | 37 |  |  |
| *tRNA-GAU* | IRB | 37 | 914 | 58 |  |  |

Table S3 Information on intron-containing genes in the chloroplast genome of *F. przewalskii*.

| **Gene** | **Location** | **Exon I (bp)** | **Intron I (bp)** | **Exon II (bp）** | **Intron II (bp)** | **Exon III (bp)** |
| --- | --- | --- | --- | --- | --- | --- |
| *ycf3* | LSC | 124 | 738 | 230 | 709 | 159 |
| *ClpP* | LSC | 71 | 769 | 294 | 584 | 250 |
| *tRNA-UUU* | LSC | 38 | 2554 | 36 |  |  |
| *tRNA-CGA* | LSC | 32 | 671 | 60 |  |  |
| *AtpF* | LSC | 160 | 767 | 410 |  |  |
| *rpoC1* | LSC | 432 | 783 | 1623 |  |  |
| *tRNA-UAA* | LSC | 35 | 533 | 50 |  |  |
| *tRNA-AAU* | LSC | 34 | 584 | 60 |  |  |
| *rpl2* | IRA | 394 | 673 | 428 |  |  |
| *NdhB* | IRA | 775 | 647 | 758 |  |  |
| *tRNA-GAU* | IRA | 36 | 916 | 57 |  |  |
| *tRNA-UGC* | IRA | 37 | 810 | 36 |  |  |
| *NdhA* | SSC | 553 | 1035 | 539 |  |  |
| *tRNA-UGC* | IRB | 38 | 808 | 37 |  |  |
| *tRNA-GAU* | IRB | 37 | 914 | 58 |  |  |

Table S4 Information on intron-containing genes in the chloroplast genome of *F. delavayi*.

| **Gene** | **Location** | **Exon I (bp)** | **Intron I (bp)** | **Exon II (bp）** | **Intron II (bp)** | **Exon III (bp)** |
| --- | --- | --- | --- | --- | --- | --- |
| *ycf3* | LSC | 124 | 738 | 230 | 709 | 159 |
| *ClpP* | LSC | 71 | 770 | 294 | 585 | 250 |
| *tRNA-UUU* | LSC | 38 | 2553 | 36 |  |  |
| *tRNA-CGA* | LSC | 32 | 671 | 60 |  |  |
| *AtpF* | LSC | 160 | 769 | 410 |  |  |
| *rpoC1* | LSC | 432 | 778 | 1623 |  |  |
| *tRNA-UAA* | LSC | 35 | 533 | 50 |  |  |
| *tRNA-AAU* | LSC | 34 | 584 | 60 |  |  |
| *rpl2* | IRA | 394 | 673 | 428 |  |  |
| *NdhB* | IRA | 775 | 647 | 758 |  |  |
| *tRNA-GAU* | IRA | 36 | 916 | 57 |  |  |
| *tRNA-UGC* | IRA | 37 | 810 | 36 |  |  |
| *NdhA* | SSC | 553 | 1043 | 539 |  |  |
| *tRNA-UGC* | IRB | 38 | 808 | 37 |  |  |
| *tRNA-GAU* | IRB | 37 | 914 | 58 |  |  |

Table S5 Information on intron-containing genes in the chloroplast genome of *F. sinica*.

| **Gene** | **Location** | **Exon I (bp)** | **Intron I (bp)** | **Exon II (bp）** | **Intron II (bp)** | **Exon III (bp)** |
| --- | --- | --- | --- | --- | --- | --- |
| *ycf3* | LSC | 126 | 738 | 228 | 709 | 159 |
| *clpP* | LSC | 71 | 770 | 294 | 585 | 250 |
| *tRNA-UUU* | LSC | 38 | 2552 | 36 |  |  |
| *tRNA-CGA* | LSC | 32 | 669 | 60 |  |  |
| *atpF* | LSC | 158 | 767 | 412 |  |  |
| *rpoC1* | LSC | 432 | 777 | 1623 |  |  |
| *tRNA-UAA* | LSC | 35 | 533 | 50 |  |  |
| *tRNA-AAU* | LSC | 34 | 584 | 60 |  |  |
| *rpl2* | IRA | 394 | 673 | 431 |  |  |
| *ndhB* | IRA | 777 | 647 | 756 |  |  |
| *tRNA-GAU* | IRA | 36 | 916 | 57 |  |  |
| *tRNA-UGC* | IRA | 37 | 810 | 36 |  |  |
| *ndhA* | SSC | 553 | 1035 | 539 |  |  |
| *tRNA-UGC* | IRB | 38 | 808 | 37 |  |  |
| *tRNA-GAU* | IRB | 37 | 914 | 58 |  |  |

Table S6 Summary of repeat sequences and SSRs in four *Fritillari*a species.

| **Species** | ***F.unibracteata*** | ***F.przewalskii*** | ***F.delavayi*** | ***F.sinica*** |
| --- | --- | --- | --- | --- |
| SSR loci (N) | 75 | 77 | 72 | 76 |
| P1ªloci (N) | 27 | 29 | 23 | 27 |
| P2ᵇloci (N) | 6 | 7 | 7 | 7 |
| P3ᶜloci (N) | 34 | 35 | 34 | 35 |
| P4 ^d^loci (N) | 8 | 6 | 7 | 6 |
| P5 ^e^loci (N) | 0 | 0 | 1 | 1 |
| Total number | 212 | 212 | 211 | 212 |
| LSC | 125 | 124 | 122 | 124 |
| SSC | 37 | 38 | 39 | 38 |
| IR | 50 | 50 | 50 | 50 |

ᵃsingle-nucleotide SSRs, ᵇdouble-nucleotide SSRs, ᶜthree-nucleotide SSRs, ^d^four-nucleotide SSRs, ^e^five-nucleotide SSRs
